# iRhom2 regulates HMGB1 secretion to modulate inflammation and hepatocyte senescence in an in vitro model of ischemia-reperfusion injury

**DOI:** 10.1038/s41419-025-08256-x

**Published:** 2026-01-07

**Authors:** Matteo Calligaris, Riccardo Perriera, Claudia Carcione, Vitale Miceli, Margot Lo Pinto, Rosalia Busà, Giandomenico Amico, Matteo Bulati, Caterina Amato, Duilio Pagano, Pier Giulio Conaldi, Simone Dario Scilabra, Massimo Pinzani, Giovanni Zito

**Affiliations:** 1 Università degli Studi di Udine, Udine, Italy; 2 IRCCS ISMETT, Palermo, Italy; 3 Ri.MED Foundation, Palermo, Italy; 4 UPMC Italy, Palermo, Italy

**Keywords:** Mechanisms of disease, Extracellular signalling molecules

## Abstract

Ischemia-reperfusion injury (IRI) represents a major challenge in liver transplantation, driving acute dysfunction and contributing to long-term allograft rejection. This process triggers a robust inflammatory response, leading to hepatocyte damage, senescence, and impaired liver regeneration. While the underlying mechanisms remain incompletely understood, increasing evidence highlights macrophage-derived signaling as a pivotal driver of hepatocyte fate during IRI. Here, we identify iRhom2 as a key regulator of immune-mediated liver injury, orchestrating macrophage-driven inflammation and hepatocyte senescence. iRhom2 is known to modulate the secretion of multiple cytokines by macrophages, yet its specific contribution to IRI-driven hepatocyte senescence has not been fully elucidated. We reveal a significant upregulation of iRhom2 in IRI+ reperfused allografts, particularly in Kupffer cells and monocyte-derived macrophages. Functional characterization in iRhom2-deficient macrophages revealed reduced ER stress, preserved mitochondrial function, and attenuated apoptosis, indicating a protective role against IRI-induced cellular damage. Proteomic profiling further uncovers iRhom2-dependent secretion of inflammatory mediators, with HMGB1 emerging as a critical damage-associated molecular pattern (DAMP) molecule in this context. Notably, HMGB1 release occurs independently of TACE catalytic activity, suggesting an alternative unexplored regulatory mechanism. Furthermore, co-culture experiments confirm that macrophage-derived HMGB1 directly induces senescence of human induced pluripotent stem cell-derived hepatocytes (hiPSC-Heps) under in vitro IRI condition, driving the up-regulation of key senescence markers and disrupting cell cycle dynamics. Strikingly, HMGB1 neutralization enhances hepatocyte viability and mitigates senescence, underscoring its pathogenic role. Additionally, HMGB1 knockdown in macrophages protects hepatocytes, though p21 expression remains unaffected, hinting at additional senescence pathways. Our findings establish iRhom2 as a central orchestrator of macrophage-driven hepatocyte dysfunction in IRI and suggest that targeting the iRhom2-HMGB1 axis could represent a promising therapeutic strategy to improve post-transplant liver recovery and long-term graft survival.

## Introduction

Liver transplantation is the primary and often the only effective treatment for end-stage liver disease, a condition that is becoming an increasingly significant cause of mortality worldwide. As transplantation rates rise, so does the urgency of addressing associated risks, particularly acute and chronic rejection, which compromise graft survival and patient outcomes [1]. Among the major contributors to graft dysfunction, ischemia-reperfusion injury (IRI) stands out as a critical factor [2, 3]. IRI occurs during the transplantation process, involving two distinct phases: ischemia, when the organ is deprived of oxygen during retrieval, and reperfusion, where oxygen restoration paradoxically exacerbates cellular damage [4, 5]. This multifaceted process initiates a cascade of events, including ATP depletion, reactive oxygen species (ROS) generation, calcium dysregulation, and endoplasmic reticulum stress (ERS), all of which converge to drive tissue injury and inflammation [3, 6].

Recent advancements have shed light on the role of inactive rhomboid protein 2 (iRhom2) as a critical regulator of cellular processes underlying IRI. Best known for its regulation of tumor necrosis factor-alpha (TNFα) converting enzyme (TACE) trafficking and TNFα release [7, 8], iRhom2 has also been implicated in ERS, calcium homeostasis, and lipid deposition. In particular, it promotes inositol trisphosphate receptor (IP3R)-mediated calcium release, which enhances ERS-induced mitochondrial dysfunction and membrane depolarization [9]. Additionally, the induction of iRhom2 by palmitate in macrophages and cardiomyocytes induces ERS and promotes macrophages to generate inflammatory factors, significantly influencing obesity-induced cardiomyopathy [10]. Together, these studies suggest a functional link between the ERS response, iRhom2, and IRI, although details regarding the mechanism and physiological significance remain scarce.

Here, we show that iRhom2 plays a crucial role in the progression of liver IRI. Analysis of liver transplanted patients showed significantly elevated iRhom2 expression in individuals with severe IRI, correlating with worse clinical outcomes. We found that iRhom2 ablation in immune cells prevents ERS and mitochondrial depolarization, mitigating IRI-associated damages. Using an in vitro IRI model with M1- macrophages, we demonstrated that iRhom2 is crucial for High-mobility group box 1 (HMGB1) secretion under ischemic conditions. HMGB1 has emerged as a key mediator of IRI in liver transplants [11]. We found that HMGB1 triggers hepatocyte senescence and worsens energy depletion, as evidenced by reduced ATP levels and altered senescent markers. Importantly, blocking HMGB1 signaling effectively reversed hepatocyte senescence and restored energy homeostasis, underscoring the specificity of iRhom2-dependent HMGB1-mediated effects in the context of IRI.

## Material and methods

### Meta-analysis of orthotopic liver transplants (OLT) and iRhom2 expression in transcriptomic databases

The RNA sequencing data analyzed in this study were retrieved from the NCBI Gene Expression Omnibus (GEO) and are accessible under accession numbers GSE87487 and GSE151648 [11]. To assess iRhom2 expression in OLT patients, transcript per million (TPM) values were compared between pre- and post-reperfusion samples using the calculation POST(TPM) - PRE(TPM) and further analyzed according to IRI status. Additionally, iRhom2 transcript levels were examined at both pre- and post-reperfusion across all 48 orthotopic allografts.

To investigate iRhom2 expression across different liver cell types, we utilized data from the Human Liver Atlas (https://www.livercellatlas.org/).

### Isolation of monocytes and differentiation in M1- macrophages

Human monocytes were obtained from 10 healthy volunteers. Buffy coats from these donors were provided by the Transfusion Center of the Ospedale “Civico” in Palermo. All donors gave written informed consent for the use of their blood for research purposes at the time of donation. The study was conducted in accordance with the Declaration of Helsinki. The study was reviewed and approved by the local Ethics Committee of ISMETT (Istituto Mediterraneo per i Trapianti e Terapie ad Alta Specializzazione) under approval code IRRB/05/24. CD14^+^ monocytes from human peripheral blood mononuclear cells (PBMC) were isolated as previously described [12–14]. M1- macrophage differentiation was performed according to the protocol established by our group [14] and Tarique and co-workers [15].

### Cell cultures

CD14^+^-derived M1- primary macrophages were cultured in RPMI 1640 medium supplemented with 10% FBS, 100 U/mL penicillin, 100 μg/mL streptomycin (Gibco Invitrogen), 5% Sodium Pyruvate and 10 mM HEPES (Euroclone, Pero MI, Italy). Human induced pluripotent stem cells (hiPSCs) were purchased from ATCC (Manassas, VA, USA). They were cultured in Essential 8 Flex Medium Kit (Thermo Fisher, Waltham, MA, USA), according to the manufacture’s recommendations. iPSC-derived hepatocytes (hiPS-Heps) were obtained by using the STEMdiff™ Hepatocyte Kit from Stem Cell Technologies (Vancouver, BC, Canada), according to the protocol suggested by the company. Briefly, iPSCs were seeded as single cells and 24 h later were induced to obtain definitive endoderm, hepatic progenitors and finally mature hepatocytes. Mature hepatocytes were cultured in STEMdiff™ Hepatic Medium for downstream experimental analysis. Human primary hepatocytes (Lot #HUM 4111 and #HUM4242) were purchased from Lonza Biosciences (Basel, Switzerland). Cells were handled and seeded according to the manufacturer’s protocols and cultivated in HBM hepatocyte culture medium (Lonza Biosciences). All the cell cultures were performed in standard conditions at 37 °C and 5% CO_2_.

### In vitro protocol of ischemia/reperfusion

In vitro IRI protocol on M1- primary macrophages and THP-1-derived macrophages was performed as previously described [14]. For co-culture IRI, wild type, iRhom2 and HMGB1-deficient M1- macrophages were differentiated in 12 mm or 6.5 mm Transwell permeable supports (ThinCerts – TC Inserts, Greiner bio-one, Kresmunster, Austria). Twenty-four hours prior to IRI, iPSC-Heps and THLE-2 cells were seeded in 12-well or 24-well plates according to experimental necessities. Right before cold ischemia, the inserts were added to the wells with iPSC-Heps and THLE-2 hepatocytes. Cold ischemia was performed in a Hypothermosol preservation medium (Sigma Aldrich, St Louis, MO, USA), while the reperfusion was done in a cell-type specific growth medium. At the experimental endpoint, hiPSC-Heps and THLE-2 cells have been harvested according to the assay to be performed.

### ROS detection by flow cytometry

Intracellular ROS levels were quantified using the cell-permeant fluorescent probe 2’,7’-dichlorofluorescin diacetate (DCFDA, Sigma-Aldrich). NTC and iRhom2 KO THP-1-derived macrophages were plated and differentiated as previously described [14] in a 12-well plate, followed by exposure to the IRI protocol. For each timepoint, the cells were washed with PBS and incubated with 10 μM DCFDA diluted in serum-free medium at 37 °C for 30 min in the dark. After incubation, cells were washed, harvested, and resuspended in FACS buffer (PBS supplemented with 1% FBS and 1 mM EDTA). ROS-associated fluorescence was immediately analyzed by flow cytometry (BD FACSCelesta) using excitation at 488 nm and emission detection in the FITC channel (530/30 nm). A minimum of 10,000 events per sample were collected. Data were analyzed using FlowJo software (TreeStar), and results were expressed as % of ROS+ cells. ROS values were normalized to control conditions and compared across experimental groups.

## Results

### iRhom2 expression increases in orthotopic liver transplanted (OLT)-IRI^+^ patients

**Fig. 1:**
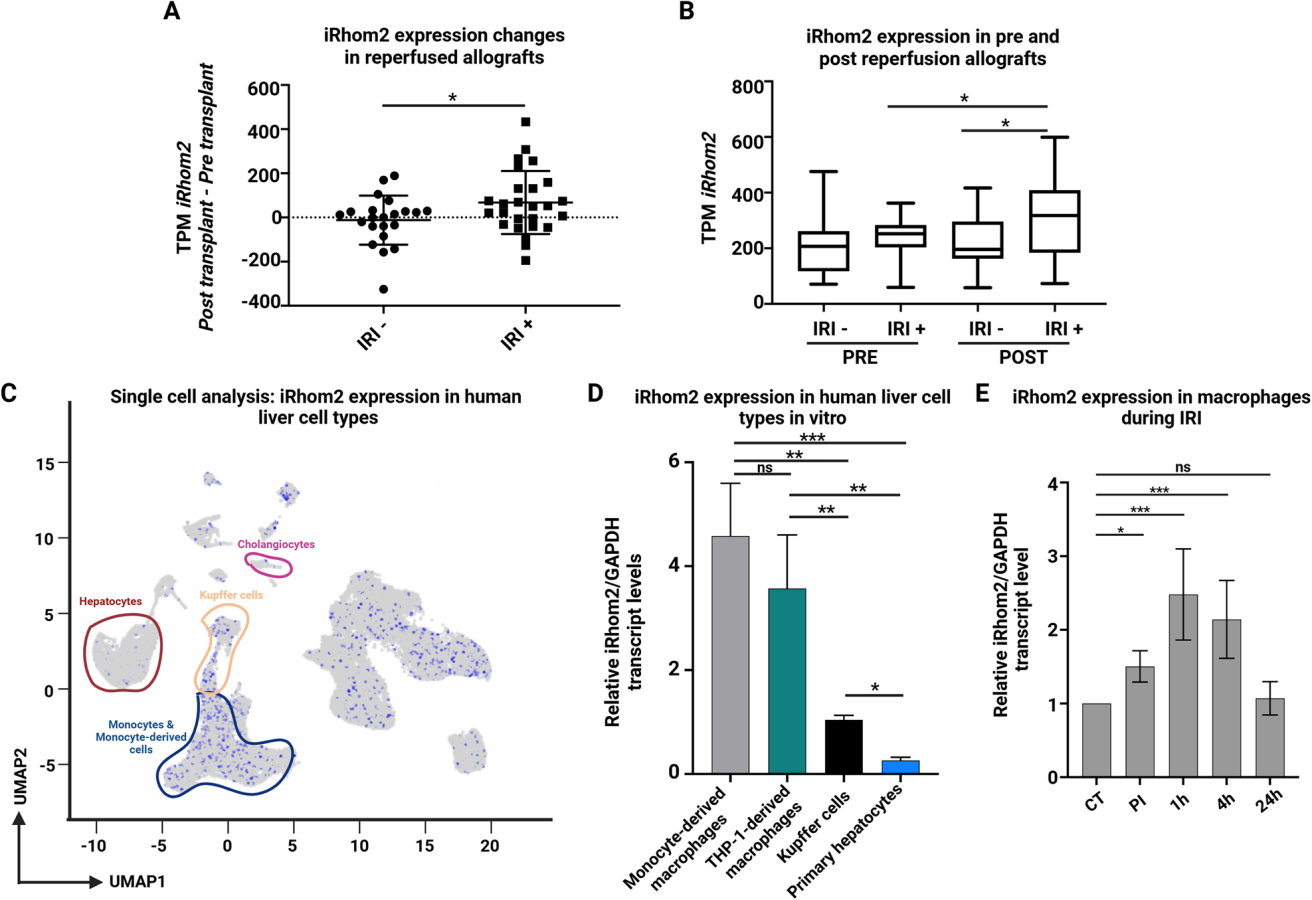
iRhom2 expression increased in OLTs and primary M1-macrophages undergoing IRI. **A** Transcript per million (TPM) of iRhom2 pre-reperfusion was subtracted from the TPM of iRhom2 post-reperfusion for every patient and shown as an individual dot. The data were grouped according to IRI occurrence (IRI+ and IRI−). **B** TPM of iRhom2 in patients pre- and post-reperfusion and clustered according to IRI occurrence were presented as Tukey box-and-whisker plots. **C** UMAP plot showing iRhom2 expression in the different liver cell types. Data obtained from repository GSE192742 (https://livercellatlas.org). **D** iRhom2 gene expression analysis in different cell types associated with liver IRI (*n* = 3). **E** Gene expression analysis of iRhom2 transcript in reperfused M1-primary macrophages at different timepoints (*n* = 7). **A** student unpaired *t*-test, **B**, **D** Ordinary one-way ANOVA with FDR correction Benjamini, Krieger and Yekutieli, **E** Ordinary one-way ANOVA with Tukey’s multiple comparison test (*p < 0.05; **p < 0.01; ***p < 0.001; ns not significant). Created in BioRender. Zito, G. (2025) https://BioRender.com/344dqqm.

To investigate the potential involvement of iRhom2 in liver IRI, we examined its expression dynamics in relation with the progression of the injury. We leveraged transcriptomic data from the GSE87487 and GSE151648 repositories, encompassing two independent transcriptomic studies with 48 orthotopic liver transplant (OLT) biopsy samples (22 IRI− and 26 IRI+; [11, 16]). Our analysis revealed a significant upregulation of iRhom2 in IRI+ reperfused allografts compared to IRI− samples (Fig. 1A), suggesting a potential role in injury exacerbation. Interestingly, pre-transplant (PRE) allograft biopsies exhibited comparable iRhom2 transcript levels across both groups (Fig. 1B, left), whereas post-transplant (POST) biopsies showed a marked increase in iRhom2 expression exclusively in IRI+ patients (Fig. 1B, right). These findings indicate iRhom2 may actively contribute to liver injury progression.

To identify the main cellular sources of iRhom2, we examined single-cell RNA-sequencing data from the liver cell atlas (GSE192742,https://livercellatlas.org). iRhom2 was predominantly expressed in Kupffer cells (KCs), monocytes, and monocyte-derived macrophages, with minimal expression in hepatocytes and cholangiocytes (Fig. 1C). These findings were validated in our own primary human liver cell populations, confirming highest expression in monocyte-derived macrophages (Fig. 1D, Supplementary Fig. S1A), consistent with an immune-mediated role for iRhom2. As prolonged ischemia depletes KCs and recruits monocyte-derived macrophages to sustain inflammation [17–19], we next investigated iRhom2 expression in an in vitro IRI model using M1-polarized macrophages [14]. iRhom2 levels increased early after reperfusion (1 h and 4 h), then returned to baseline at 24 h (Fig. 1E, Supplementary Fig. S1B), mirroring the transient inflammatory wave observed during IRI.

Collectively, these findings indicate that iRhom2 is dynamically regulated in immune cells during liver IRI and may contribute to the amplification of immune-driven injury post-transplantation.

### iRhom2 deficiency mitigates ER stress and mitochondrial dysfunction in IRI

**Fig. 2:**
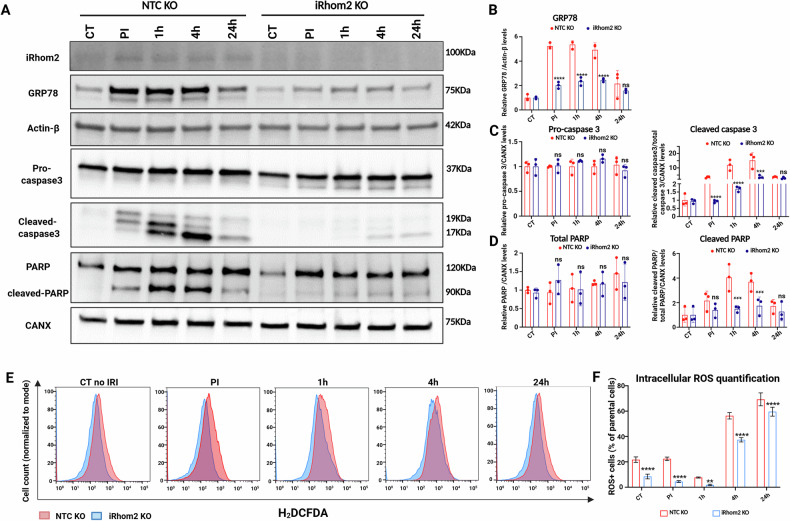
iRhom2 ablation improves cell recovery and ER stress in M1- macrophages during IRI. **A** Representative immunoblot and relative quantification (**B**–**D**) of iRhom2, GRP78, Pro-caspase3, cleaved-caspase 3, PARP, and cleaved-PARP, in NTC or iRhom2 KO THP-1 derived macrophages during in vitro IRI at different time points. Actin-β and Calnexin (CANX) were used as loading controls (*n* = 3). **E**, **F** ROS detection and relative quantification in NTC or iRhom2 KO THP-1 derived macrophages during in vitro IRI at different time points. **B**–**D**, **F** Multiple unpaired *t* tests (*p < 0.05; **p < 0.01; ***p < 0.005; ****p < 0.001; ns not significant). Created in BioRender. Zito, G. (2025) https://BioRender.com/vuvm400.

To define the role of iRhom2 in IRI-induced damage, we compared THP-1-derived M1 macrophages lacking iRhom2 (KO) to non-targeting controls (NTC) [20]. As in primary macrophages, iRhom2 expression was upregulated upon polarization, and KO cells showed reduced TNFα secretion without changes in mRNA levels (Supplementary Fig. S1C), indicating post-transcriptional regulation. Since IRI strongly activates ERS, we examined mitochondrial and ER responses in both cell lines under ischemia and reperfusion. NTC cells exhibited marked ATP depletion and mitochondrial mass loss during ischemia, with partial recovery at 24 h (Supplementary Fig. S1D, E, black line). Mitochondrial depolarization peaked 4 h post-reperfusion (Supplementary Fig. S1F, black line), accompanied by robust glucose-regulated protein 78 (GRP78) induction (ERS marker), and cleavage of caspase-3 and PARP (Fig. 2A–D), consistent with ER stress-induced apoptosis. By contrast, iRhom2 KO cells showed a faster ATP recovery and better maintenance of mitochondrial membrane potential after reperfusion (Supplementary Fig. S1D–F). GRP78 was still induced but at lower levels compared to NTC cells, suggesting dampened ER stress (Fig. 2A, B). Apoptotic markers, including cleaved caspase-3 and PARP, were also reduced in KO cells (Fig. 2A, C, D), indicating diminished cell death. To investigate whether reduced oxidative stress underlies these effects, we quantified intracellular ROS using DCFDA and flow cytometry. iRhom2-deficient cells displayed significantly lower ROS levels than controls (Fig. 2E, F), supporting a role for iRhom2 in amplifying oxidative injury. Overall, these data suggest that iRhom2 contributes to IRI-induced mitochondrial and ER dysfunction, in part by enhancing ROS accumulation. Its absence confers cytoprotective effects by limiting stress signaling and apoptosis, positioning iRhom2 as a potential therapeutic target in liver IRI.

### iRhom2 controls HMGB1 secretion during IRI in a TACE-catalytically independent manner

**Fig. 3:**
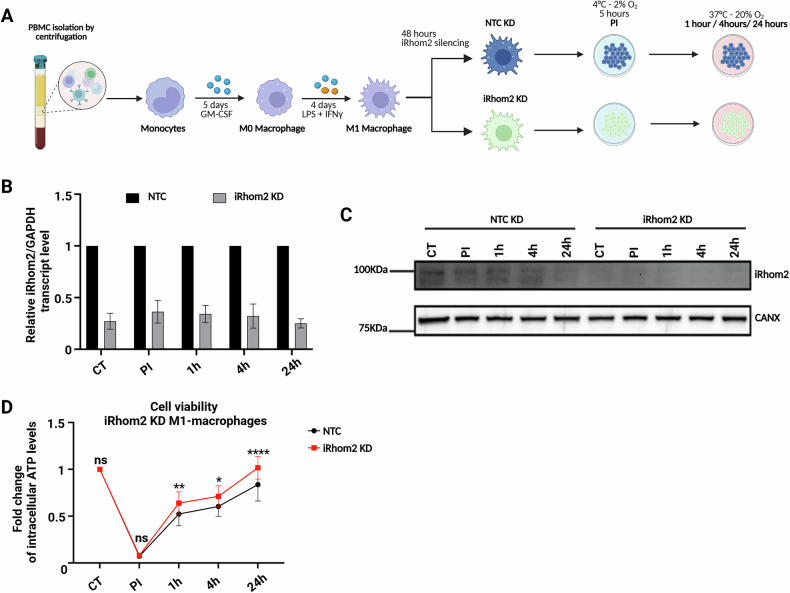
iRhom2 inhibition regulates M1-macrophage viability. **A** Schematic representation of the modified in vitro IRI protocol established for the study. **B** Gene expression analysis of iRhom2 transcript in reperfused NTC or iRhom2 KD primary macrophages at different timepoints (*n* = 4). **C** Representative immunoblot showing the levels of iRhom2 at different time points upon in vitro reperfusion in NTC or iRhom2 KD macrophages. Calnexin (CANX) was used as loading control. **D** Intracellular ATP in NTC (black line) or iRhom2 KD (red line) macrophages during in vitro IRI at different time points. Data are represented as % of ATP activity in the experimental conditions vs. control (CT) not induced to ischemia (*n* = 4). **B** Multiple unpaired *t*-tests. **D** Two-way ANOVA with Sidak correction (*p < 0.05; **p < 0.01; ****p < 0.0001; ns not significant). Created in BioRender. Zito, G. (2025) https://BioRender.com/j39w730.

Our findings suggest that iRhom2 regulates ER stress and apoptosis during in vitro IRI. To further investigate its role in primary macrophages, we applied an in vitro IRI protocol to CD14⁺ monocyte-derived M1 macrophages subjected to iRhom2 knockdown (KD) (Fig. 3A). Efficient silencing was confirmed at both transcript and protein levels (Fig. 3B, C), along with reduced TNFα transcription and secretion (Supplementary Fig. S2A), validating functional inhibition.

**Fig. 4:**
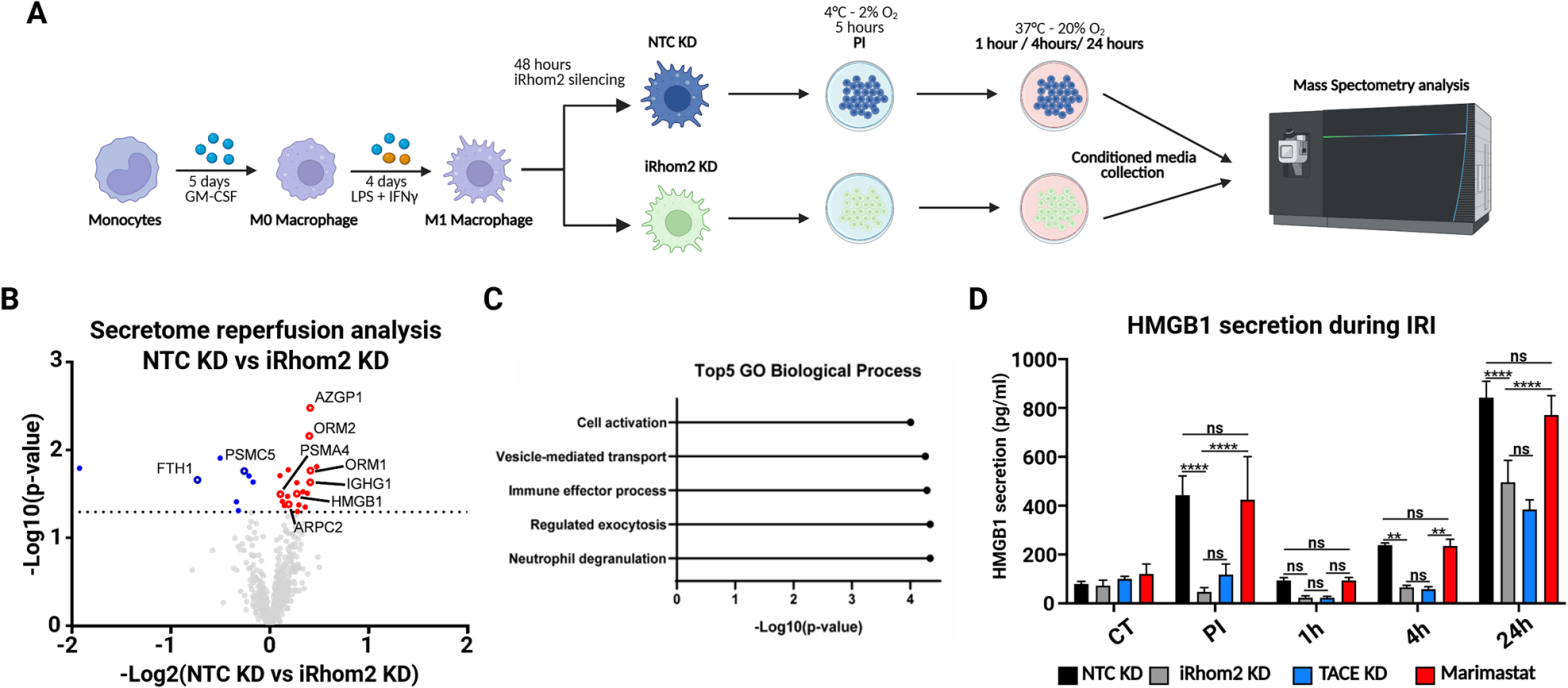
iRhom2 controls inflammation and HMGB1 secretion in a TACE-independent manner during IRI. **A** Schematic representation of the modified in vitro IRI protocol established for the study. **B** Volcano plot showing the -Log10 of p-values versus the log2 of protein ratio between iRhom2 KD and NTC KD macrophages at 4 h reperfusion (*n* = 4). Statistical significance was set at *p* < 0.05 and log2 (fold change) >0.5 was represented as black dashed lines. Secreted proteins significantly reduced in iRhom2 KD macrophages are displayed as red-filled dots, unchanged and less abundant proteins as transparent gray dots. **C** Top 5 functional GOBP for proteins decreased in iRhom2 KD macrophages secretome analysis. **D** ELISA assay showing HMGB1 protein quantification in the conditioned medium (CM) of reperfused NTC KD, iRhom2KD, TACE KD and Marimastat-treated M1 primary macrophages at different IRI time points (*n* = 3). **D** Two-way ANOVA with Sidak correction (**p < 0.01; ****p < 0.001; ns not significant). Created in BioRender. Zito, G. (2025) https://BioRender.com/1ih7lgh.

We then examined whether iRhom2 KD influences macrophages viability under IRI condition. Consistent with results in THP-1 cells, iRhom2 KD primary macrophages showed improved metabolic recovery, as indicated by sustained ATP levels throughout reperfusion, despite similar values during the post-ischemic phase (PI) (Fig. 3D). To gain mechanistic insights into how iRhom2 deficiency influences macrophage function, we performed a label-free quantitative proteomic profiling of the macrophage secretome under metabolic and ER stress (Fig. 4A). The volcano plot in Fig. 4B showed a broad dysregulation of secreted proteins in iRhom2 KD macrophages, with a notable reduction in key mediators associated with IRI progression at 4 h post-reperfusion (red dots). Among these, the secretion of XPNPEP1, ORM1, LRG1, and HMGB1 - all of which are known hallmarks of IRI identified in human liver biopsies [21] – was significantly reduced in iRhom2 KD macrophages, reinforcing the hypothesis that iRhom2 downregulation confers protection against IRI-induced damage. GO analysis confirmed downregulation of pathways involved in neutrophil degranulation, exocytosis, and immune effector functions (Fig. 4C; Supplementary Table S1), suggesting an attenuated pro-inflammatory profile in iRhom2-deficient macrophages. Given the well-established role of the alarmin HMGB1 in sterile inflammation and tissue injury, we specifically examined its secretion dynamics in iRhom2 KD macrophages. ELISA confirmed significantly reduced extracellular HMGB1 in iRhom2 KD macrophages at PI, 4 h, and 24 h (Fig. 4D), while intracellular protein and mRNA levels remained unchanged (Supplementary Fig. S2B, C), indicating regulation at the level of secretion. TACE knockdown also suppressed HMGB1 release. However, this effect is attributed to TACE’s role in stabilizing iRhom2 rather than its catalytic activity, as treatment with the TACE inhibitor Marimastat did not alter HMGB1 release (Fig. 4D). Indeed, it has been shown that TACE KD resulted in the rapid degradation of iRhom2 [22]. In contrast, TNFα secretion was strongly suppressed upon iRhom2, TACE KD, and Marimastat treatment, confirming the expected TACE-catalytic dependency (Supplementary Fig. S2D).

Taken together, our high-resolution shotgun proteomics analysis reveals that iRhom2 critically modulates the secretion of IRI-associated markers in primary macrophages. Specifically, we identify HMGB1 as an iRhom2-dependent secretory protein whose release occurs independently of TACE catalytic activity.

### iRhom2 regulates hepatocytes senescence during IRI

**Fig. 5:**
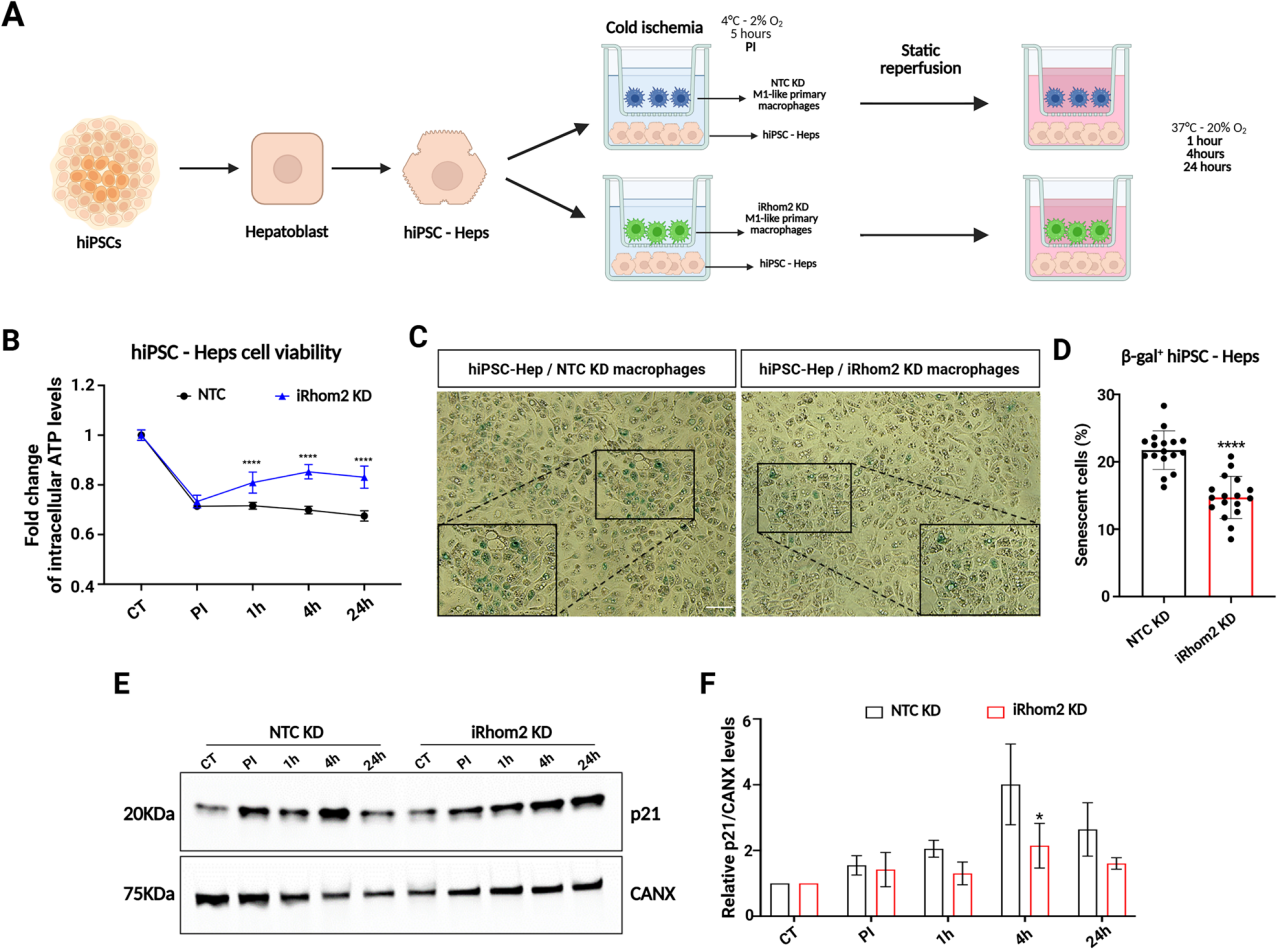
iRhom2 ablation in primary macrophages reduced senescence of hiPSC-derived hepatocytes during reperfusion. **A** Schematic representation of the co-cultured NTC or iRhom2 KD macrophages with hiPSC-derived hepatocytes and subjected to IRI. **B** Intracellular ATP quantification in hiPSC-Heps co-cultured with NTC (black line) or iRhom2 KD (red line) macrophages during IRI at different time points (*n* = 12). **C** Representative pictures of hiPSC-Heps co-cultured with NTC or iRhom2 KD macrophages for 24 h of reperfusion and stained for β-Galactosidase (Scale bar: 100 μm). **D** Senescence quantification of β-Galactosidase+ hiPSC-Heps co-cultured with NTC or iRhom2 KD macrophages at 24 h of reperfusion. **E** Representative immunoblot of p21 in hiPSC-Heps co-cultured with NTC or iRhom2 KD macrophages and subjected to IRI. p21 protein level was analyzed at all the different reperfusion timepoints. Calnexin (CANX) was used as loading control. **F** p21 protein quantification from hiPSC-Heps co-cultured with NTC or iRhom2 KD macrophages and subjected to IRI (*n* = 3). **B** Two-way ANOVA with Sidak correction, **D**, **F** Multiple unpaired t-tests (*p < 0.05; **p < 0.005; ****p < 0.0001; ns not significant). Created in BioRender. Zito, G. (2025) https://BioRender.com/lyqjlhy.

It has been already proven that metabolic stress, including IRI, ERS, and ATP depletion, trigger cellular senescence [23–25]. Our findings suggest that iRhom2 plays a central role in modulating ERS, mitochondrial dysfunction and ATP depletion, implicating a possible link between energy stress and senescence-associated secretory phenotype in primary macrophages. Consistently, our proteomic analysis revealed that iRhom2 KD impairs the secretion of several IRI-associated markers including the senescence-associated alarmin HMGB1. To investigate the role of macrophage-derived factors in the hepatocyte responses to IRI, we employed a co-culture system integrating NTC KD or iRhom2 KD monocyte-derived primary macrophages and hiPSC-Heps, to elucidate the paracrine function of iRhom2 in senescence regulation (Fig. 5A). hiPSC-Heps were generated using an established differentiation protocol requiring 21 days of culture to obtain mature hepatocytes that closely resemble adult primary hepatocytes morphologically (Supplementary Fig. 3A, left panel). The success of the differentiation protocol was validated through immunofluorescence staining for hepatocyte-specific markers (CK18, A1AT and Albumin), along with gene expression analysis (HNF4A, Albumin) and albumin secretion assay (Supplementary Fig. 3A- right panel, and 3B), confirming the suitability of this model for studying hepatocyte-macrophage interactions in the context of IRI. Using this co-culture system, we observed that hiPSC-Heps exhibited a significantly faster recovery during static reperfusion when cultured with iRhom2 KD macrophages compared to those co-cultured with NTC KD cells (Fig. 5B). Notably, hepatocytes co-cultured with iRhom2 KD macrophages displayed a substantial reduction in senescence, as evidenced by a decreased number of β-galactosidase+ cells (Fig. 5C, D). To further confirm this observation, we assessed the expression of p21 protein, a key regulator of cell cycle arrest/senescence [26–28]. Immunoblot analysis demonstrated a marked reduction in p21 expression in hiPSC-Heps co-cultured with iRhom2 KD macrophages during static reperfusion (Fig. 5E, F), suggesting that iRhom2-dependent macrophage signaling plays a crucial role in promoting hepatocyte senescence under IRI conditions. Importantly, this senescence phenotype was not associated with apoptosis, as cleaved caspase-3 levels remained unchanged between hiPSC-Heps co-cultured with NTC KD and iRhom2 KD macrophages (Supplementary Fig. 4). Altogether, these findings highlight a novel paracrine function of iRhom2 in regulating hepatocyte senescence phenotype during IRI.

### HMGB1 regulates hepatocyte senescence during IRI

**Fig. 6:**
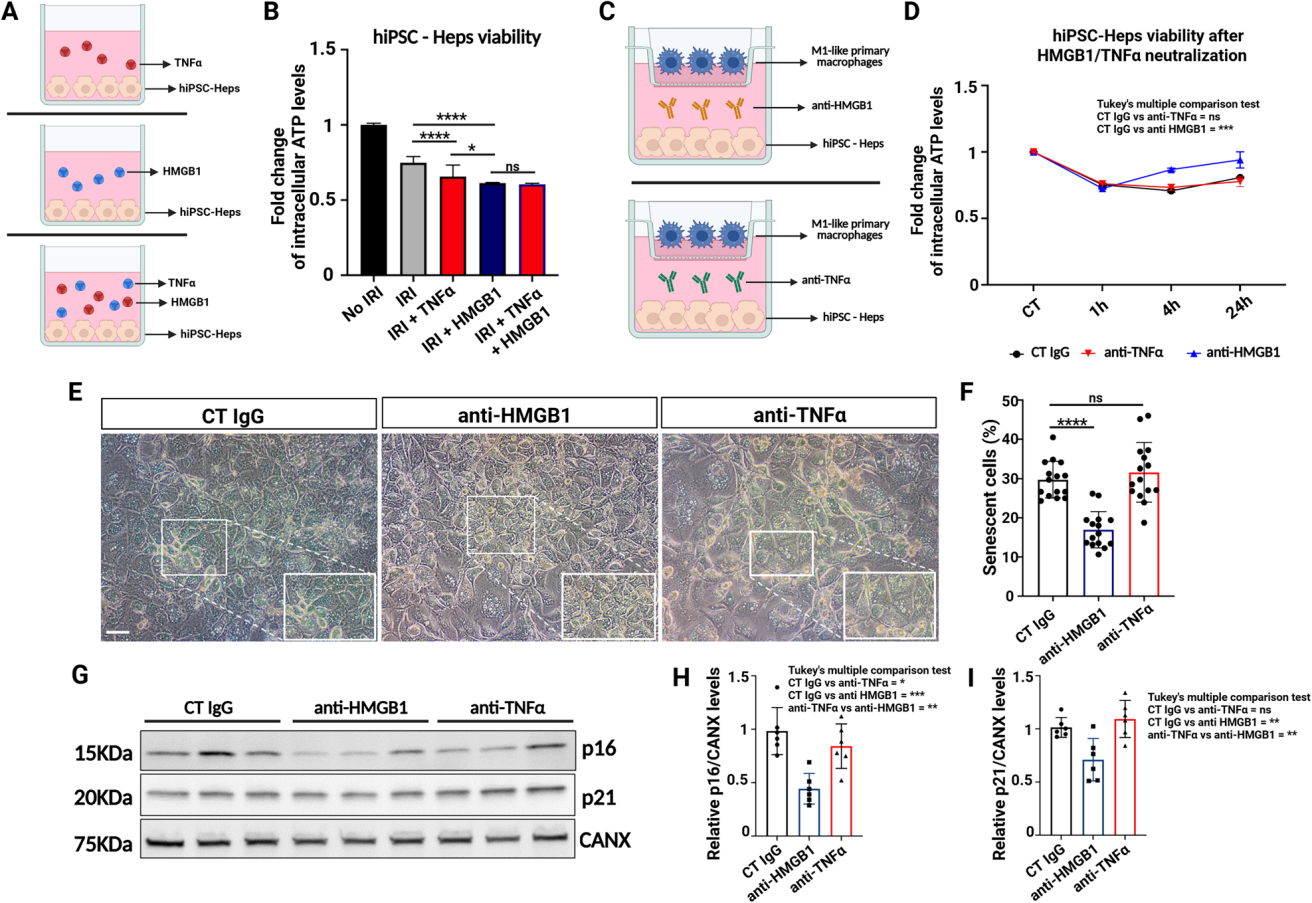
HMGB1 is required to induce hepatocyte senescence during IRI. **A** Schematic representation showing hiPSC-Heps hepatocytes treated with soluble HMGB1, TNFα, or a combination of both during reperfusion. **B** Intracellular ATP quantification in hiPSC-Heps treated with soluble HMGB1, TNFα or a combination of both during reperfusion (*n* = 4). Data are represented as % of ATP in the experimental conditions vs. control (not induced to IRI). **C** Schematic representation of the co-cultured M1- primary macrophages with hiPSC-Heps and subjected to IRI with neutralizing antibody anti-HMGB1 or anti-TNFα during reperfusion. **D** Intracellular ATP quantification in hiPSC-Heps treated with neutralizing antibody anti-HMGB1 or anti-TNFα during reperfusion (*n* = 4). **E** Representative pictures of hiPSC-Heps co-cultured with activated M1- primary macrophages for 24 h of reperfusion and stained for β-Galactosidase (Scale bar: 100 μm). **F** Senescence quantification of β-Galactosidase+ hiPSC-Heps co-cultured with activated macrophages at 24 h of reperfusion. **G** Representative immunoblot of p16 and p21 in hiPSC-Heps treated with neutralizing antibody anti-HMGB1 or anti-TNFα and subjected to IRI. p16 and p21 protein levels were analyzed at 24 h reperfusion. Calnexin protein (CANX) was used as loading control. **H** p16 and p21 protein quantification from hiPSC-Heps treated with neutralizing antibody anti-HMGB1 or anti-TNFα and subjected to IRI. p16 and p21 proteins were analyzed at 24 h reperfusion. **D**, **H**, **I** One-way ANOVA with Tukey correction, **F** unpaired *t*-tests (*p < 0.05; ****p < 0.005; ns not significant). Created in BioRender. Zito, G. (2025) https://BioRender.com/ahmrf54.

Our findings support the hypothesis that iRhom2 in macrophages regulates a paracrine mechanism that induces hepatocyte senescence during IRI. However, the specific factors mediating this effect remain to be identified. Among the IRI markers downregulated in iRhom2 KD macrophages, we focused on HMGB1, given its well-established extracellular role in modulating senescence. To investigate this hypothesis, we subjected hiPSC-Heps and THLE-2 hepatocytes to IRI, and treated them with recombinant HMGB1 during reperfusion. As a control, we used TNFα, a cytokine known to be secreted in an iRhom2-dependent manner and reported to induce senescence under certain conditions [29] (Fig. 6A and Supplementary Fig. S5A). Notably, hepatocytes treated with HMGB1 exhibited significantly reduced viability compared to untreated control cells subjected to IRI alone. In contrast, TNFα treatment had only a minor impact on intracellular ATP levels (Fig. 6B, S5B). Interestingly, when cells were treated with both recombinant HMGB1 and TNFα, ATP depletion mirrored that observed in the HMGB1-only condition, suggesting that HMGB1, rather than TNFα, is the principal macrophage-derived factor contributing to ATP depletion during reperfusion.

To further explore whether HMGB1 secretion is required for hepatocyte senescence during IRI, we performed neutralization experiments using anti-HMGB1 antibodies in a co-culture system of hiPSC-Hep and THLE-2 cells with M1-primary macrophages subjected to IRI (Fig. 6C and S5C). As a control, we used anti-TNFα antibodies. Strikingly, anti-HMGB1 treatment significantly improved metabolic activity, as evidenced by increased intracellular ATP levels, while anti-TNFα treatment had no significant effect (Fig. 6D, Supplementary Fig. S5D). Consistently, β-galactosidase staining revealed a marked reduction in senescence in hepatocytes treated with anti-HMGB1, whereas anti-TNFα treatment did not significantly alter senescence levels (Fig. 6E, F and Supplementary Fig. S5E, F). Furthermore, p16 and p21 and p53 expression – key regulators of cellular senescence - were significantly reduced in hepatocytes treated with anti-HMGB1, while anti-TNFα treatment had no substantial effect (Fig. 6G–I and Supplementary Fig. S5G, H). Collectively, these results indicate that HMGB1 – but not TNFα - is the predominant mediator of hepatocyte senescence in our in vitro IRI model.

### Secreted HMGB1 regulates hepatocytes senescence during IRI

**Fig. 7:**
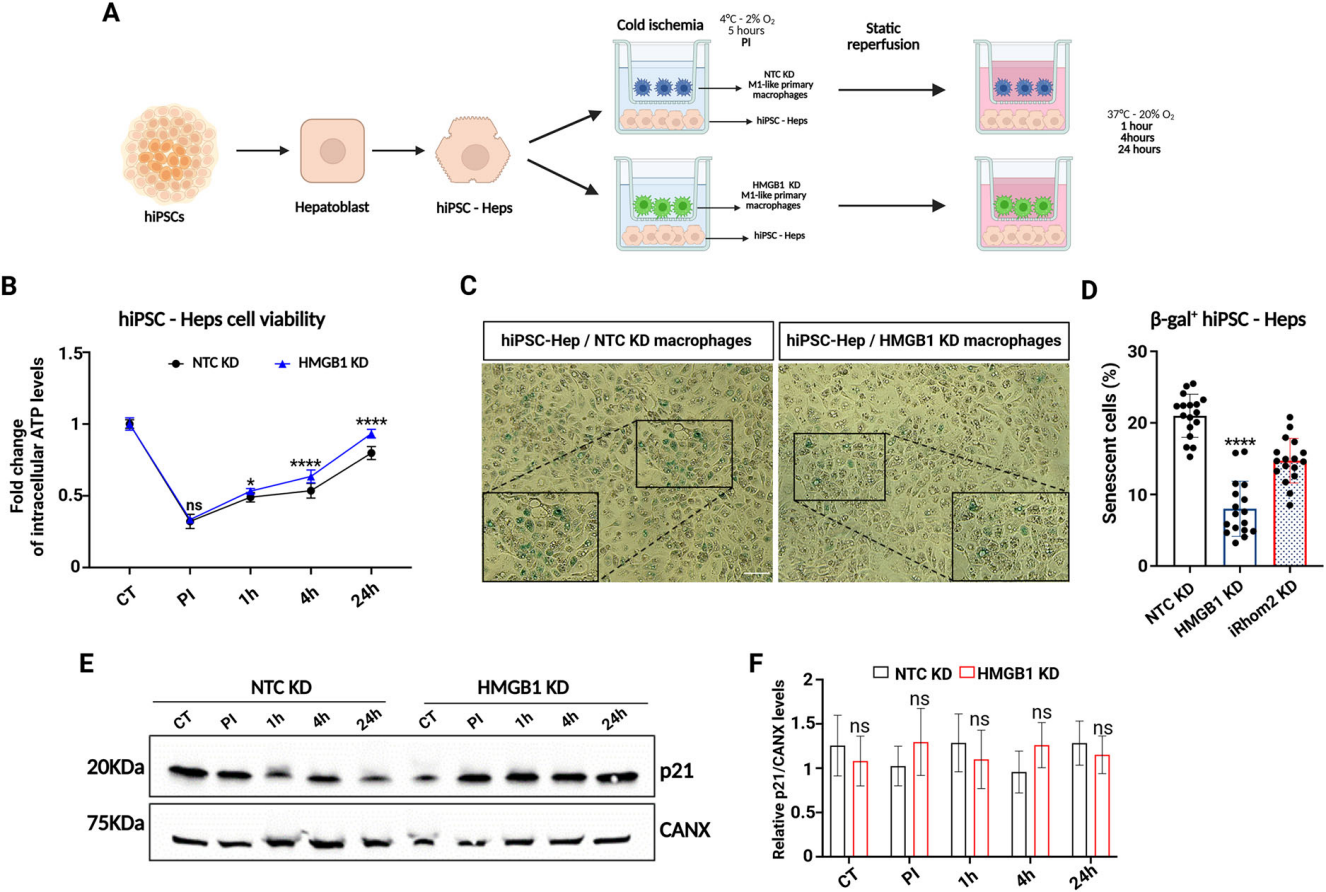
HMGB1 genetic ablation is sufficient to reduce hepatocytes senescence during in vitro IRI. **A** Schematic representation of the co-cultured NTC or iRhom2 KD macrophages with hiPSC-derived hepatocytes and subjected to IRI. **B** Intracellular ATP quantification in hiPSC-Heps co-cultured with NTC (black line) or HMGB1 KD (blu line) primary macrophages during IRI at different time points (*n* = 12). **C** Representative pictures of hiPSC-Heps co-cultured with NTC or HMGB1 KD macrophages for 24 h of reperfusion and stained for β-Galactosidase (Scale bar: 100 μm). **D** Senescence quantification of β-Galactosidase+ hiPSC-Heps co-cultured with NTC or HMGB1 KD macrophages at 24 h of reperfusion. iRhom2 KD histogram derives from data already shown in Fig. 5D. **E** Representative immunoblot of p21 in hiPSC-Heps co-cultured with NTC or HMGB1 KD macrophages and subjected to IRI. p21 protein level was analyzed at all the different reperfusion timepoints. Calnexin (CANX) was used as loading control. **F** p21 protein quantification from hiPSC-Heps co-cultured with NTC or HMGB1 KD macrophages and subjected to IRI (*n* = 3). **B** Two-way ANOVA with Sidak correction, **D**, **F** Multiple unpaired *t*-tests (*p < 0.05; ****p < 0.0001; ns not significant). Created in BioRender. Zito, G. (2025) https://BioRender.com/olphvlp.

To determine whether HMGB1 is required for paracrine activation of hepatocyte senescence during IRI, we genetically silenced HMGB1 in M1- primary macrophages (HMGB1 KD), and co-cultured them with hiPSC-Heps and THLE-2 cells under in vitro IRI conditions (Fig. 7A and Supplementary Fig. S6A, B). Consistent with the effects observed in the iRhom2 KD–hepatocyte co-culture, viability assays demonstrated that hepatocytes co-cultured with HMGB1 KD macrophages exhibited a faster recovery during reperfusion compared to those co-cultured with control macrophages (Fig. 7B and Supplementary Fig. S6C). Importantly, β-galactosidase assays confirmed that HMGB1 inhibition reduced hepatocyte senescence, further supporting its role as a key paracrine factor in this process (Fig. 7C, D and Supplementary Fig. S6D, E). However, we did not observe any changes in p21/p53 regulation, suggesting that other mechanisms are involved in fully activating the senescence pathway (Fig. 7E, F). Overall, these results indicate that while paracrine HMGB1 can induce hepatocyte senescence, it is not essential for activating p21/p53-dependent senescence during IRI.

## Discussion

Liver transplantation remains the only curative option for end-stage liver disease [30], yet ischemia-reperfusion injury (IRI) continues to be a major challenge, affecting graft function and long-term survival. Despite advancements in surgical techniques, organ preservation, and immunosuppressive regimens, no targeted therapies currently exist to prevent or attenuate IRI [31–36], contributing to early allograft dysfunction, chronic rejection, and limiting the use of marginal grafts, thereby reducing the available donor pool [30, 37].

IRI is a complex, biphasic process involving tightly interconnected cellular and molecular mechanisms. During the ischemic phase, oxygen and nutrient deprivation disrupt cellular homeostasis, resulting in ATP depletion, redox imbalance, ERS and mitochondrial dysfunction. Reperfusion, while essential for tissue re-oxygenation, paradoxically intensifies injury through the sudden burst of ROS and an overwhelming inflammatory response that amplifies tissue damage and exacerbates hepatocellular injury [38].

Targeting ERS has shown promise in mitigating other IRI-associated damages. Pharmacological inhibition of ERS has been associated with improved autophagic flux, reduced lipid accumulation, and decreased apoptosis in preclinical models [39]. However, complete suppression of ERS may compromise protective adaptive responses, leading to protein misfolding, toxic aggregate accumulation, and exacerbated apoptosis. Therefore, a more refined approach is needed to modulate specific pathways, particularly those involved in inflammatory and stress-related responses.

In the last decade, iRhom2 has emerged as a key regulator of TACE trafficking and TNFα release [7, 8], and its iRhom2 inhibition has been explored as a potential therapeutic strategy for inflammatory diseases [40–43]. More recently, iRhom2 has been implicated in ER stress-induced cell death [9] and inflammatory responses triggered by ERS [10]. Thus, given its involvement in different cellular processes, iRhom2 represents an attractive target for IRI research and a potential candidate for post-transplantation therapies.

To our knowledge, this is the first study to investigate iRhom2 in the context of liver transplantation. Leveraging transcriptomic data from 48 orthotopic liver transplant (OLT) patients in the Sosa et al. dataset—one, if not the only, of the few datasets including paired pre- and post-ischemia samples—we found significant upregulation of iRhom2 transcripts in IRI+ patients post-reperfusion [11, 16]. Since iRhom2 is highly expressed in the peripheral blood cells rather than liver parenchyma [44], its increased expression in IRI+ patients likely stems from immune cells, triggering inflammatory mechanisms that drive immune cell infiltration - a histological hallmark of IRI severity [16].

To further investigate iRhom2’s role, we subjected M1-macrophages genetically ablated to an in vitro IRI protocol. This resulted in reduced mitochondrial damage, as indicated by increased intracellular ATP levels during reperfusion, and a modest activation of intrinsic mitochondrial apoptosis. Previous studies have shown that iRhom2-deficient cells exposed to ER stressors exhibit minimal changes in unfolded protein response (UPR) activation, but display alterations in ER stress-induced cell death [9]. Interestingly, our findings suggest that IRI-induced UPR regulation is disrupted in iRhom2-deficient cells, as evidenced by reduced GRP78 activation. This highlights, in a complex injury model like IRI, a unique role for iRhom2 in linking ER stress to oxidative and inflammatory pathways — an interaction not observed in classical ER stress models. In addition to these findings, our data demonstrate that iRhom2 deficiency significantly reduces intracellular ROS levels during reperfusion, supporting the hypothesis that iRhom2 contributes to mitochondrial dysfunction by amplifying oxidative stress. Given that iRhom2 is localized to the ER and regulates inflammatory signaling, its effect on mitochondrial homeostasis appears to be mediated through the downstream attenuation of ER stress and inflammatory cascades. The combined impact on ER and mitochondrial integrity likely contributes to the reduced secretion of DAMPs, reinforcing the idea that iRhom2 coordinates a broader stress response during hepatic IRI. While different studies support such mechanisms [45–47], this mechanistic hypothesis requires further validation in the in vitro IRI model.

A particularly striking and novel aspect of our study is the identification of iRhom2 as an indirect regulator of HMGB1 secretion, independently of its canonical role in TACE activation. HMGB1 is a key mediator of sterile inflammation [48, 49] and contributes to hepatocyte senescence and impaired liver regeneration post-transplant [50]. Given the known role of ROS as an upstream trigger of HMGB1 release during reperfusion injury [51–53], and the subsequent activation of NF-κB-dependent pro-inflammatory pathways, it is conceivable that the iRhom2-HMGB1 axis identified in our study also intersects with oxidative stress mechanisms. This hypothesis requires further investigation, as iRhom2 dependent ROS-driven HMGB1 secretion either via exosome secretion or autophagy could represent an additional layer of regulation contributing to hepatocyte senescence and graft dysfunction [46, 54]. Proteomic profiling of iRhom2-deficient macrophages revealed additional secreted proteins altered in iRhom2-deficient macrophages, such as XPNPEP1 and ORM1. While HMGB1 was prioritized in our functional studies due to its established role in senescence and strong downregulation in the iRhom2 KD secretome, these other factors may act independently or synergistically to influence hepatocyte fate. Functional validation of these candidates is warranted to delineate the broader consequences of iRhom2-dependent secretome remodeling. During IRI, macrophages actively secrete HMGB1, which binds to TLR4 and TLR9 receptors, initiating inflammatory cascades that drive hepatocyte senescence, apoptosis, and impaired regeneration [55]. This mechanism underlies acute graft dysfunction and long-term complications, including fibrosis and chronic rejection [56–59]. Our in vitro co-culture IRI model shows that reduced HMGB1 secretion from iRhom2-deficient macrophages was sufficient to prevent hepatocyte senescence, highlighting iRhom2-HMGB1 axis as a critical regulator of energy depletion and hepatocyte survival [60, 61]. Notably, pharmacological neutralization of HMGB1—but not TNFα inhibition—rescued the senescent phenotype, underscoring the specificity of the iRhom2-HMGB1 axis. While TNFα has been implicated in senescence in other contexts [29], our data suggest that HMGB1 plays the predominant role in macrophage-induced hepatocyte injury in this model. We realize that this approach does not fully recapitulate the complexity of liver IRI in vivo, as it lacks several non-parenchymal cell types and the liver’s 3D structure. However, it remains a useful tool to investigate specific macrophage-hepatocyte interactions and key molecular mechanisms under ischemic conditions. Although direct evidence from in vivo models is currently lacking, our study identifies a novel iRhom2-dependent mechanism regulating HMGB1 secretion from macrophages during hepatic IRI. The involvement of both iRhom2 and HMGB1 in inflammatory processes and liver injury is well supported by existing literature [7, 41, 62, 63], suggesting that the iRhom2-HMGB1 axis may similarly contribute to tissue damage and repair in vivo. Future validation in animal models of hepatic IRI is essential to confirm the translational relevance of this pathway and assess its therapeutic potential.

**Fig. 8:**
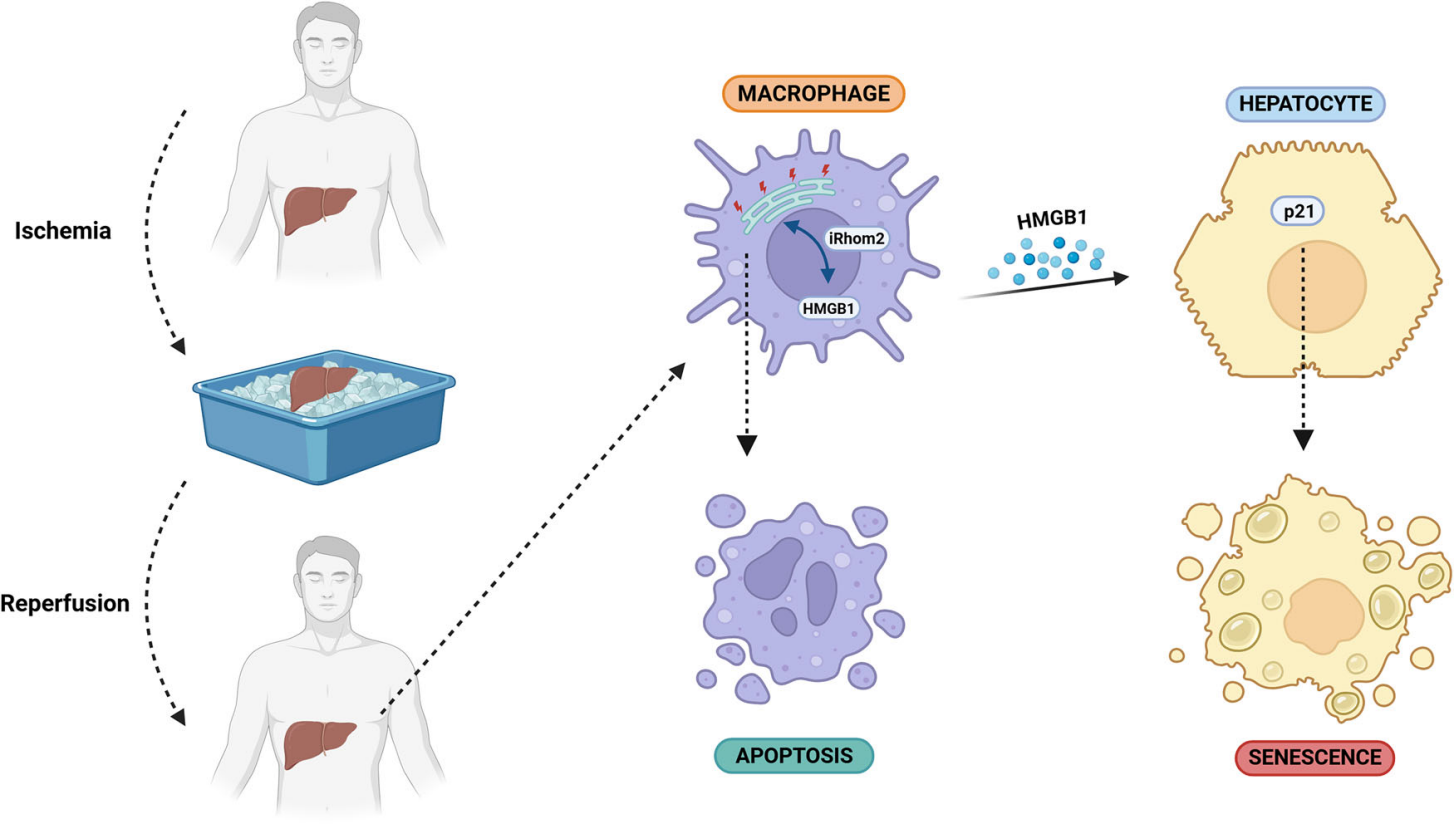
Proposed model describing the paracrine role of iRhom2 in regulating hepatocyte senescence during in vitro liver IRI. Created in BioRender. Zito, G. (2025) https://BioRender.com/o6p2h8v.

In conclusion, our study identifies iRhom2 as a key mediator of hepatic IRI, orchestrating ER stress, oxidative stress, HMGB1 secretion, and hepatocyte senescence. (Fig. 8). Targeting iRhom2 may provide a more selective and effective therapeutic strategy than global inhibition of HMGB1 or ER stress, which could disrupt essential physiological processes. Future research should focus on pharmacologic modulation of iRhom2 activity as a means to mitigate IRI-induced injury and improve long-term outcomes in liver transplantation.

## Supplementary information

Supplementary information

Supplementary FIles_ Original WB

## Data Availability

Data set including the proteomic analysis are available in Proteome Exchange (https://proteomecentral.proteomexchange.org/ui?search=PXD046469). All other data are available upon reasonable request.
