# Supplementary material for: iRhom2 regulates HMGB1 secretion to modulate inflammation and hepatocyte senescence in an in vitro model of ischemia-reperfusion injury
Source: Cell Death Dis. 2026 Jan 7;17(1):7. doi: 10.1038/s41419-025-08256-x (PMC12780076; doi:10.1038/s41419-025-08256-x)
Supplement: Supplementary file 1 — Supplementary information [file 41419_2025_8256_MOESM1_ESM.pdf]

iRhom2 regulates HMGB1 secretion to modulate inflammation and hepatocyte senescence in an in vitro model of ischemia-reperfusion injury.

Matteo Calligaris[1**], Riccardo Perriera[2**], Claudia Carcione[3], Vitale Miceli[2], Margot Lo Pinto[2], Rosalia Busà[2], Giandomenico Amico[3], Matteo Bulati[2], Caterina Amato[2], Duilio Pagano[4], Pier Giulio Conaldi[2*], Simone Dario Scilabra[3], Massimo Pinzani[2] and Giovanni Zito[2*].

[1]Department of Medicine (DMED), Università degli Studi di Udine, Udine, Italy.

[2]Research Department, Istituto Mediterraneo per i Trapianti e Terapie ad alta specializzazione - IRCCS ISMETT, Via Ernesto Tricomi 5, Palermo, Italy.

[3] Ri.MED Foundation, via Ernesto Tricomi 5, Palermo, Italy

[4]Abdominal Surgery and Organ Transplant Unit, Department for the Treatment and Study of Abdominal Diseases and Abdominal Transplantation, Istituto Mediterraneo per i Trapianti e Terapie ad alta specializzazione (IRCCS-ISMETT), Via Ernesto Tricomi 5, Palermo, Italy.

*Correspondence: Giovanni Zito (GZ), gzito@ismett.edu

** These authors have contributed equally to this work and share first authorship

The supplementary information correlated to the manuscripts includes:

1- Supplementary figures S1-S6
2- Supplementary tables S1-S3
3- Supplementary Material and Methods
4- Supplementary References

Supplementary Figures and Figure Legends

Supplementary Figure S1

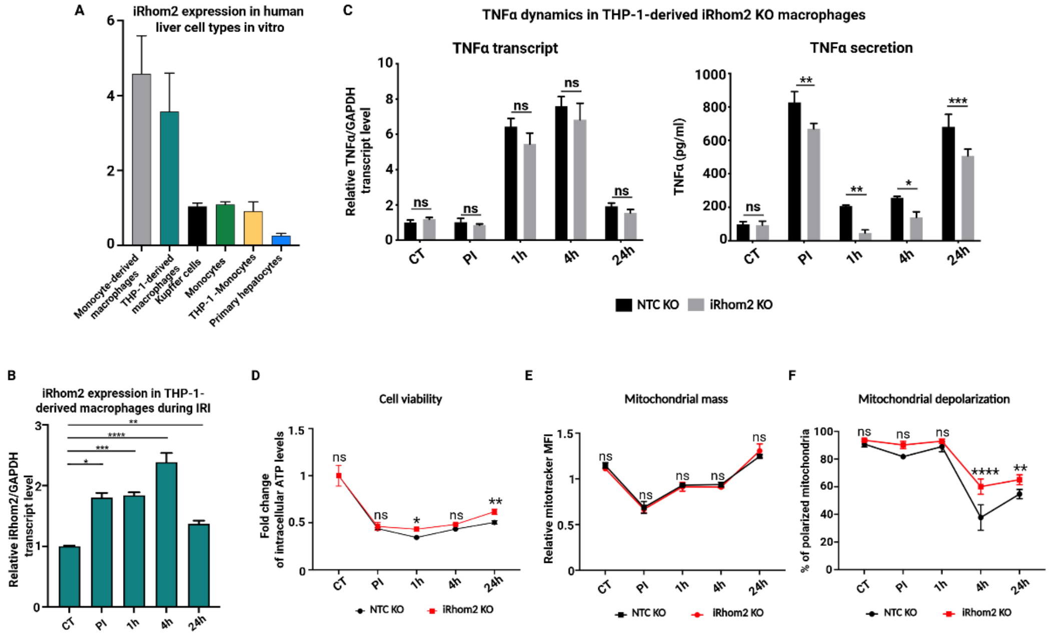

Supplementary Figure S1: (A) iRhom2 gene expression analysis in different liver cell types associated with liver IRI (n = 3). (B) Gene expression analysis of iRhom2 transcript in reperfused THP-1-derived macrophages at different time points (n = 3). (C) Left panel, gene expression analysis of TNFα transcripts in reperfused THP-1-derived NTC or iRhom2 KO macrophages at different time points (n=3); right panel, TNFα quantification in the conditioned medium of reperfused THP-1-derived NTC or iRhom2 KO macrophages at different time points (n = 3). (D) Intracellular ATP in NTC (black line) or iRhom2 KO (red line) THP-1 derived macrophages during in vitro IRI at different time points. Data are represented as % of ATP activity in the experimental conditions vs. control (CT) not induced to ischemia (n = 3). (E) Mitotracker assay to measure mitochondrial mass on NTC (black line) or iRhom2 KO (red line) THP-1 derived macrophages during in vitro IRI at different time points. Data are represented as relative mean fluorescence intensity (MFI) normalized to untreated NTC cells not induced to ischemia (n = 3). (F) Mitochondrial depolarization was assessed by JC-1 assay and data are represented as % of PE and FITC positive cells at each time-point for NTC and iRhom2KO THP-1 (n=3). For all gene expression analysis, the comparison among samples has been conducted by using the ΔΔCT method and normalized to GAPDH transcript. (A) Ordinary one-way ANOVA with FDR correction Benjamini, Krieger and Yekutieli, (* = $p < 0.05$; **= $p < 0.01$; *** = $p < 0.001$; **** = $p < 0.005$). (B-F) Statistical analysis has been calculated via two-way ANOVA with Sidak's multiple comparison test to determine differences among timepoints (* = $p< 0.05$; ** = $p < 0.005$; *** = $p < 0.0001$; ns = not significant). Created in BioRender. Zito, G. (2025) https://BioRender.com/e1n7n4j

Supplementary Figure S2

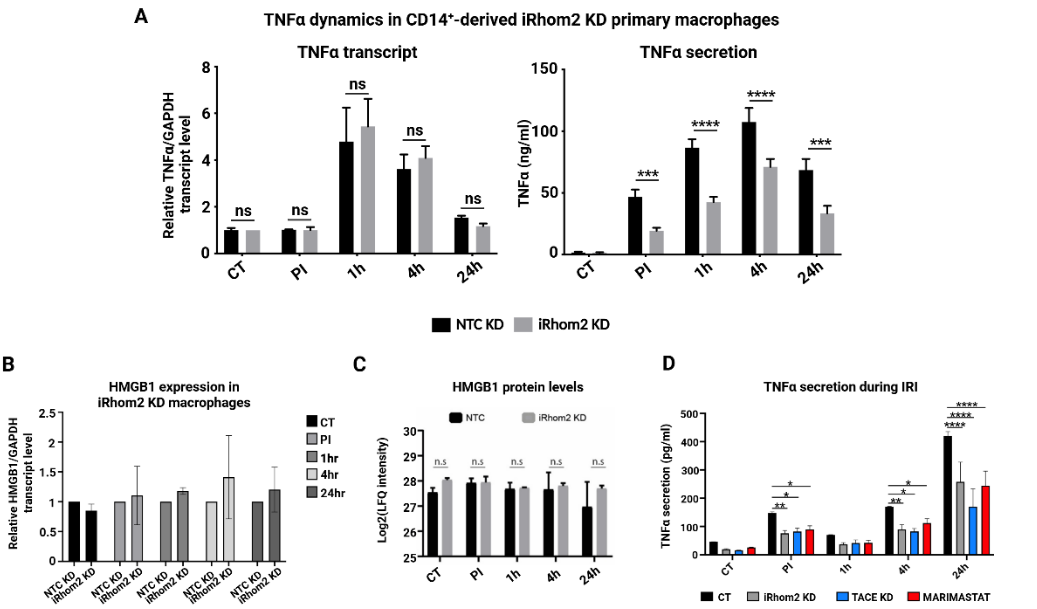

Supplementary Figure S2: (A) Left panel, gene expression analysis of TNFα transcripts in reperfused NTC or iRhom2 KD M1-like primary macrophages at different time points (n=3) Right panel, TNFα quantification in the conditioned medium of reperfused NTC or iRhom2 KD M1-like macrophages at different timepoints (n = 3). (B) Gene expression analysis of HMGB1 transcript in reperfused NTC and iRhom2 KD M1 primary macrophages at different time points. (n=3). (C) Histograms showing the log2 transformed levels of HMGB1 in the lysate of NTC and iRhom2 KD macrophages measured by mass-spectrometry and label-free quantification at different timepoints. (D) ELISA assay showing TNFα protein quantification in the conditioned medium of reperfused NTC KD, iRhom2KD, TACE KD and Marimastat-treated M1 primary macrophages at different IRI time points (n=3). For all gene expression analysis, the comparison among samples has been conducted by using the ΔΔCT method and normalized to GAPDH transcript. (A-D) Statistical analysis has been calculated via two-way ANOVA with Sidak's multiple comparison test to determine differences among timepoints (* = p< 0.05; ** = p < 0.005; *** = p < 0.0001; **** = p < 0.005; ns = not significant). Created in BioRender. Zito, G. (2025) https://BioRender.com/tl7uiit

Supplementary Figure S3

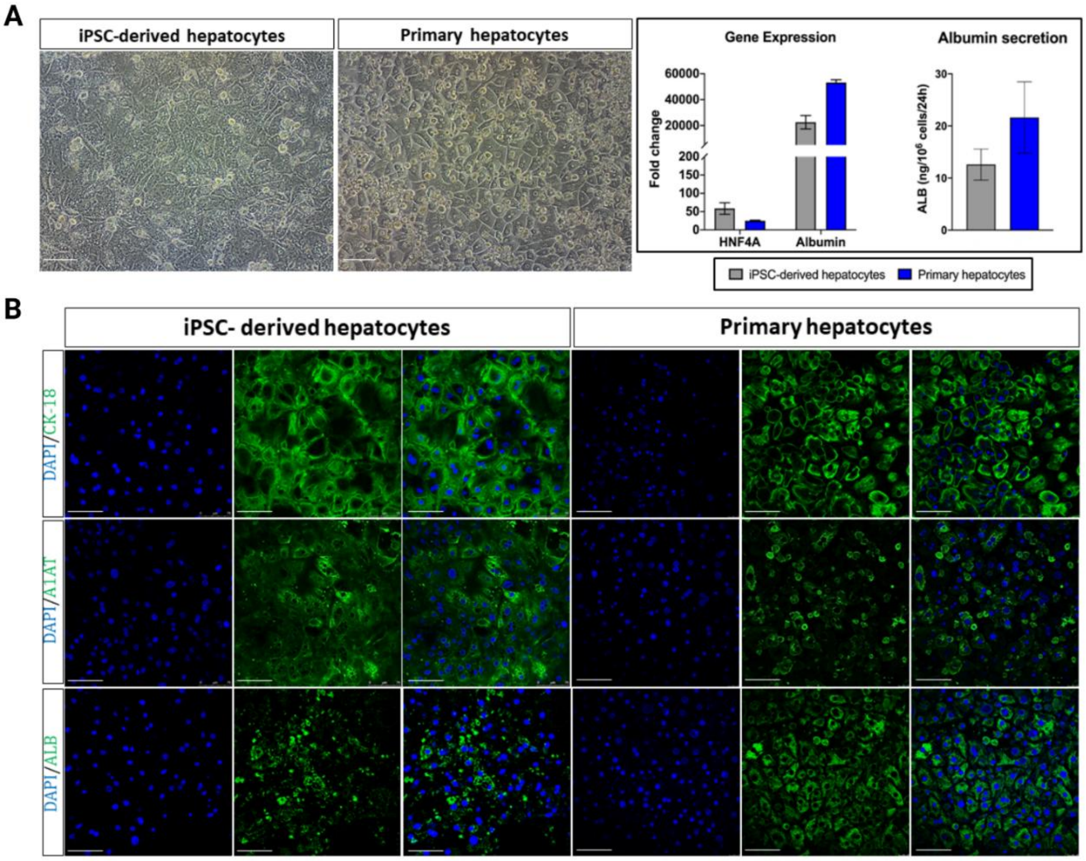

Supplementary Figure S3. Morphological and molecular characterization of hiPSC-Heps in comparison with human primary hepatocytes. (A) The left panel shows representative images of hiPSC-Heps at the end of the differentiation protocol compared to cultivated human primary hepatocytes (Scale bar: 100µm). Right panel, gene expression analysis of HNF4A and Albumin transcript in hiPSC-Heps and primary hepatocytes (n=3). The comparison was conducted using the ΔΔCT method and normalized to GAPDH transcript. In the same panel, secreted albumin quantification by ELISA assay (n=3). (B) Immunofluorescence staining for the hepatocyte markers CK18, A1AT and Albumin in hiPSC-Heps and primary hepatocytes (Scale bar: 75µm). Created in BioRender. Zito, G. (2025) https://BioRender.com/t23mfog

Supplementary Figure S4

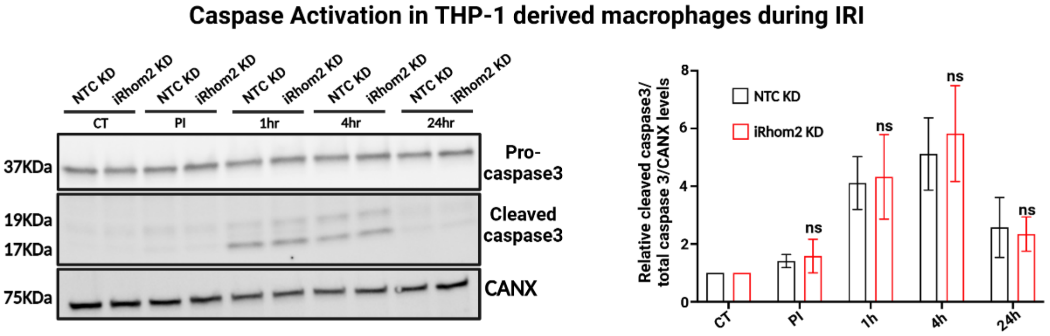

Supplementary Figure S4. Representative western blot (left panel) and relative quantification (right panel) of Pro-caspase 3 and cleaved Caspase 3 in reperfused NTC and iRhom2 KD M1 primary macrophages at different time points (n=3). Calnexin (CANX) was used as loading control. Multiple unpaired t-test. Created in BioRender. Zito, G. (2025) https://BioRender.com/hanx2tm

Supplementary Figure S5

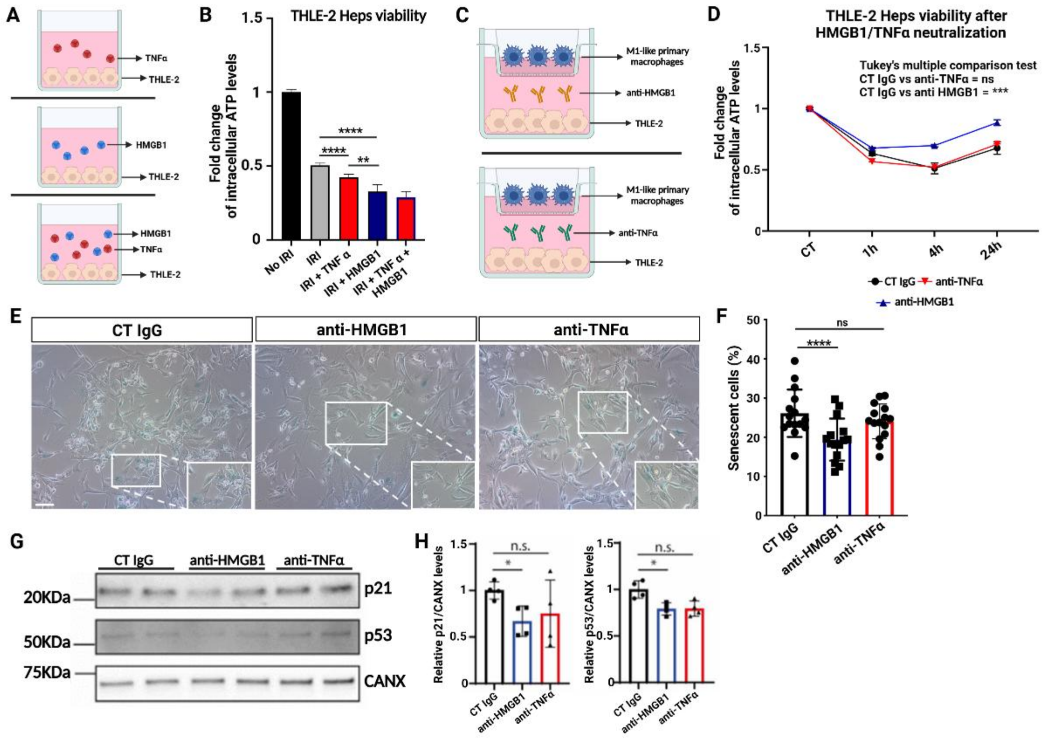

Supplementary Figure S5. (A) Schematic representation showing THLE-2 hepatocytes treated with soluble HMGB1, TNFα, or a combination of both during reperfusion. (B) Intracellular ATP quantification in THLE-2 hepatocytes treated with soluble HMGB1, TNFα or a combination of both during reperfusion (n=3). Data are represented as % of ATP in the experimental conditions vs. control (not induced to IRI). (C) Schematic representation of the co-cultured M1-like primary macrophages with THLE-2 hepatocytes and subjected to IRI with neutralizing antibody anti-HMGB1 or anti-TNFα during reperfusion. (D) Intra-cellular ATP quantification in THLE-2 hepatocytes treated with neutralizing antibody anti-HMGB1 or anti-TNFα during reperfusion (n=4). (E) Representative pictures of THLE-2 hepatocytes co-cultured with activated M1-like primary macro-phages for 24h of reperfusion and stained for β-Galactosidase (Scale bar: 100µm). (F) Senescence quantification of β-Galactosidase+ THLE-2 hepatocytes co-cultured with activated macrophages at 24h of reperfusion. (G) Representative immunoblot of p53 and p21 in THLE-2 hepatocytes treated with neutralizing antibody anti-HMGB1 or anti-TNFα and subjected to IRI. p53 and p21 protein levels were analyzed at 24h reperfusion. Calnexin protein (CANX) was used as loading control. (H) p53 and p21 protein quantification from THLE-2 hepatocytes treated with neutralizing antibody anti-HMGB1 or anti-TNFα and subjected to IRI. p53 and p21 proteins were analyzed at 24h reperfusion. (A, F, H) student unpaired t-test (* = p< 0.05; **** = p < 0.005; ns = not significant). (D) One-way ANOVA with Tukey correction. Created in BioRender. Zito, G. (2025) https://BioRender.com/17iaadi

Supplementary Figure 6.

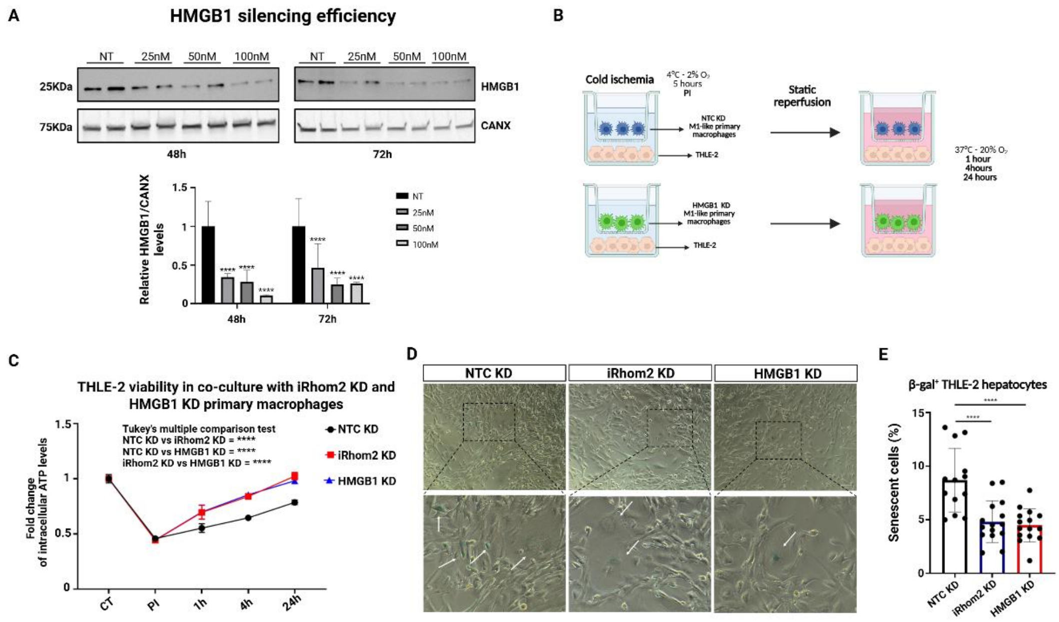

Supplementary Figure 6. (A) WB analysis and relative quantification of HMGB1 silencing efficiency in M1-like primary macrophages at 48 and 72h of different siRNA concentration treatments. Calnexin protein (CANX) was used as loading control. (B) Schematic representation of the co-cultured NTC or iRhom2 KD macrophages with THLE-2 hepatocytes and subjected to IRI. (C) Intracellular ATP quantification in THLE-2 hepatocytes co-cultured with NTC (black line), HMGB1 KD (blue line) or iRhom2 KD (red line) primary macrophages during IRI at different time points (n = 12). (D) Representative pictures of THLE-2 hepatocytes co-cultured with NTC, HMGB1 KD or iRhom2 KD macrophages for 24h of reperfusion and stained for β-Galactosidase (Scale bar: 100µm). (E) Senescence quantification of β-Galactosidase+ THLE-2 hepatocytes co-cultured with NTC, HMGB1 KD and iRhom2 KD macrophages at 24h of reperfusion. (C) Two-way ANOVA with Tukey's multiple correction, (A, E) unpaired student t-test (**** = p < 0.0001). Created in BioRender. Zito, G. (2025) https://BioRender.com/xagt42b

Supplementary Table S1: List of proteins significantly (p-value < 0.05, ratio > 0.5) decreased in the secretome of iRhom2 KD macrophages at 4h reperfusion.

Protein names	Protein ID	Gene names	Peptides	Ratio	p-value
Xaa-Pro aminopeptidase 1	Q9NQW7	XPNPEP1	14	0.95	1.53E-02
Alpha-1-acid glycoprotein 1	P02763	ORM1	12	0.83	1.70E-02
Zinc-alpha-2-glycoprotein	P25311	AZGP1	19	0.83	3.26E-03
Ig gamma-1 chain C region	P01857	IGHG1	5	0.82	2.29E-02
Alpha-1-acid glycoprotein 2	P19652	ORM2	13	0.81	6.83E-03
Leucine-rich alpha-2-glycoprotein	P02750	LRG1	9	0.77	3.06E-02
Alpha-1B-glycoprotein	P04217	A1BG	32	0.72	4.41E-02
Ceruloplasmin	P00450	CP	21	0.68	2.94E-02
Hemopexin	P02790	HPX	36	0.59	4.15E-02
Nucleosome assembly protein 1-like 1	P55209	NAP1L1	6	0.57	4.94E-02
High mobility group protein B1	Q5T7C4	HMGB1	4	0.56	3.10E-02
Rho-related GTP-binding protein RhoG	P84095	RHOG	7	0.55	2.32E-02

Supplementary Material and Methods

Cell cultures

Human epithelial hepatocyte cell line THLE-2 (ATCC, CRL-2706) was cultured as previously described [1]. The human monocytic cell line THP-1 (ATCC TIB-202, kindly provided by Dr Chiara Cipollina, Fondazione Ri.MED) was grown in complete RPMI 1640 medium supplemented with 10% FBS, 100 U/mL penicillin, 100 μg/mL streptomycin (Gibco Invitrogen), 5% Sodium Pyruvate and 10 mM HEPES (Euroclone, Pero MI, Italy). The iRhom2 KO THP1 has been previously described [2]. THLE-2 culture was performed in bronchial epithelial cell basal medium with additives (BEGM from Lonza/Clonetics Corporation, Bend, OR, USA, BEGM Bullet Kit), and supplemented with 10% Fetal Bovine Serum (FBS, Hyclone, Logan, UT, USA), 100 U/mL penicillin, 100 μg/mL streptomycin (Gibco Invitrogen, Waltham, MA, USA) and 70 ng/mL phospho-ethanolamine (Sigma Aldrich, St. Louis, MO, USA). All the cell lines were cultured at 37°C in a humidified environment containing 5% CO2.

M1-like primary macrophages silencing

After differentiation and prior to cold ischemia M1-like primary macrophages were subjected to iRhom2 and HMGB1 silencing with 50nM of an equimolar mix of 3 siRNAs for each target (HSS128594, HSS128595, HSS188104 for iRhom2; ID106695 and ID106696 for HMGB1 – Thermo Fisher Scientific) or an equimolar mix of negative control stealth siRNAs for 48 hours (Low, Med, High GC), by using Lipofectamine RNAiMAX (Thermo Fisher Scientific), and according to the manufacturer's instructions. The efficiency of knockdown was confirmed by qRT-PCR and Western blot analysis.

Cell viability assay

Intracellular ATP levels were measured to evaluate the cell viability during in vitro IRI. Cells were harvested at each time point of the IRI protocol and ATP levels have been evaluated by Cell Titer Glo 2.0 kit (Promega, Fitchburg, WI, USA) according to manufacturer's instructions. Luminescence was measured with the Spark Plate Reader (Tecan, Zurich, Switzerland), following the instructions provided by the manufacturer. Raw luminescence data were normalized to untreated cells and displayed as fold change of intracellular ATP levels.

Mitochondrial membrane potential and mass assessment via Flow Cytometry

NTC and iRhom2 KO THP-1-derived macrophages were plated and differentiated as previously described in a 12-well plate, followed by exposure to the IRI protocol. To assess mitochondrial function, cells were stained separately with JC-1 (1 µg/mL, T-3168, Thermo Fisher Scientific) and MitoTracker Deep Red (100 nM, M22426, Thermo Fisher Scientific, Waltham, MA, USA) for 15 minutes at 37°C. Following incubation, cells were washed, resuspended in PBS, and analyzed by flow cytometry according to the manufacturer's protocol. Mitochondrial membrane potential was evaluated by determining the percentage of cells exhibiting both green (monomeric JC-1, depolarized mitochondria) and red (JC-1 aggregates, polarized mitochondria) fluorescence. Mitochondrial mass was quantified based on MitoTracker Deep Red fluorescence intensity, with changes at each time point calculated as the relative mean fluorescence intensity (MFI) normalized to untreated WT cells.

Senescence associated β-Galactosidase (SA β-Gal) staining.

Senescence was evaluated by β-Gal staining according to the manufacturer's instructions (Cell Signaling). Briefly, after the co-culture protocol, transwells with THP-1 cells were removed, and THLE-2 cells were washed twice with PBS and fixed for 15 minutes with Fixative Solution. Subsequently, the cells were incubated for 6 hours at 37°C with β-Galactosidase Staining solution (prepared according to the instructions provided by the manufacturer). Five images for six biological replicates per condition were taken, and the quantification was performed as the % of β-Galactosidase + cells (positive cells/total cells x 100) per field with the Cell Counter package on Image J software.

Cytokine, inhibitor antibody and Marimastat treatments.

Recombinant TNFα (Peprotech, London, UK) and HMGB1 (R&D Systems, Minneapolis, MN, USA) were used at the concentration of 10ng/ml and 200ng/ml, respectively, during warm static reperfusion. Neutralizing TNFα and HMGB1 antibodies were obtained from Arigo Biolaboratories (Burlington, ON, Canada) and used at concentrations of 10ng/ml and 5μg/ml, respectively, during warm static reperfusion. HMGB1 release was assessed on M1-like primary macrophages in presence of 10μM Marimastat (Sigma Aldrich, St. Louis, MO, USA), a metalloprotease inhibitor, during the reperfusion phase.

RNA extraction and Gene expression analysis.

Total RNA from M1-primary macrophages was extracted by miRNAeasy (Qiagen, Hilden, Germany) according to manufacturer's instruction and 100ng of RNA was reverse transcribed using

High-Capacity cDNA Reverse Transcription Kit (Thermo Fisher Scientific). The cDNA was used for quantitative PCR (qRT-PCR) using TaqMan Expression Master Mix II (Applied Biosystem, Waltham, MA, USA) and the TaqMan probes displayed in Supplementary Table S2. Gene expression was quantified using StepOnePlus Real-Time PCR, normalized according to GAPDH expression and expressed as relative quantities compare to the corresponding control and displayed in histograms as mean values. Error bars indicate the standard deviation of biological replicates.

Supplementary Table S2: List of primers used for gene expression analysis

**Assay ID**	**Gene Symbol**	**Description**
Hs00226277_m1	RHBDF2	TaqMan® Gene Expression Assay
Hs02786624_g1	GAPDH	TaqMan® Gene Expression Assay
Hs07287366_m1	HMGB1	TaqMan® Gene Expression Assay
Hs00174128_m1	TNFα	TaqMan® Gene Expression Assay

Protein extraction and Western blot

Protein extraction was performed during the in vitro IRI protocol at each time point to analyze. Briefly, cells were washed with PBS and lysed in STET Lysis Buffer (10mM Tris-HCl, 1mM EDTA, 100mM NaCl, 1% Triton X-100, 1x Proteinase Inhibitor mixture (Sigma Aldrich), 10mM 1-10 phenanthroline (Sigma Aldrich)). Protein concentration was measured using a colorimetric 660 nm microBCA assay (Thermo Fisher Scientific) and comparable amounts of proteins were separated on 4-20% SDS/Polyacrylamide gels (Genscript, Piscataway, NJ, USA) and transferred onto poly-(vinylidene difluoride) membranes (BioRad, Hercules, CA, USA). The membranes were blocked with 5% non-fat milk (Sigma Aldrich) in PBS with 0.1% Tween and incubated with primary antibodies displayed in table 1. Bound primary antibodies were detected with peroxidase-

conjugated anti-rabbit and anti-rat secondary antibodies (Promega), by using the ECL detection system (BioRad). Protein bands were detected by a Chemidoc Image Analyser (BioRad) and their intensity quantified by ImageLab Software (BioRad). Densitometric quantification of specific proteins were normalized for their loading control and displayed in histograms as mean values ± standard deviation.

Shotgun secretome analysis by LC-MS/MS

Sample preparation for high-resolution shotgun proteomics on conditioned media from M1-like primary macrophages was done as previously described [2]. Briefly, conditioned media collected at 4 hours of reperfusion were processed using filter-aided sample preparation (FASP) with a 10 kDa Vivacon spin filter (Sartorius, Göttingen, Germany) [3]. Samples were denaturated by incubation with 50mM DTT in UA buffer (8M Urea, 100mM Tris/HCl, pH 8.5) and alkylated with 50mM iodoacetamide (IAA) in UA buffer. After reduction and alkylation, samples were digested with 0,2 µg LysC (Promega) in UC buffer (2M urea, 25mM Tris/HCl, pH 8) overnight at 37°C and to another digestion step with 0,1 µg Trypsin (Promega) for 4 hours at 37°C. The resulting peptides were desalted by stop-and-go extraction (STAGE) on reverse phase tips packed with C18 disks in-house (Empore, Sigma-Aldrich) [4] and eluted with 40 µL of a buffer containing 60% acetonitrile and 0,1% FA in mass spec grade water. Peptides were then dried by using a vacuum centrifugation and resuspended in 20 µL 0.1% FA. Peptide concentrations were measured by Nanodrop 2000 (Thermo Fisher Scientific). A total amount of 1.5 µg peptides were loaded per each sample onto a Dionex Ultimate 3000 RSLCnano LC system (Thermo Fisher Scientific), which was coupled online via a Nanospray Flex Ion Source (Thermo Fisher Scientific) to a Q-Exactive+ mass spectrometer

(Thermo Fisher Scientific). Data-dependent acquisition (DDA) was used for label-free quantification (LFQ). Raw data were analysed with the software Maxquant, version 2.0.1.0 (maxquant.org, Max-Planck Institute Munich). The MS data were searched against a reviewed canonical FASTA database of Homo sapiens from UniProt (download: November 5th 2020). Trypsin/P was defined as a protease. Standard settings were applied, with the exception of enabling the 'match between runs' option, using a match time window of 1.5 minutes, along with data normalization.

The protein LFQ reports of Maxquant were processed in Perseus [5]. The protein LFQ intensities were log2 and filtered for LFQ intensities detected in at least three biological replicates per group to be considered quantifiable, and a volcano plot was obtained with the integrated tool. A two-sided Student’s t-test was applied to check for significant changes between the groups. Further details and raw data have been deposited to the ProteomeXchange Consortium via the PRIDE partner repository with the dataset identifier PXD0464469.

Analysis of secreted proteins by ELISA

Conditioned media from reperfused cells was used for the detection, by ELISA assay, of TNFα (Human TNFA INSTANT ELISA, Thermo Fischer Scientific), and HMGB1 (Novus Biologicals, Littleton, CO, USA). ELISA assay was used also to quantify albumin release during iPSC differentiation into hepatocytes (Abnova, Taipei, Taiwan). All the assays have been performed according to the manufacturer’s protocols. Absorbance was assessed at 450nm in the Spark Plate Reader (Tecan). Protein concentration has been calculated according to the standard curve of each ELISA assay.

Immunofluorescence assay

iPSC-Heps were seeded on laminin-coated rounded coverslips and differentiated according to manufacturer's instruction (STEMdiffTM Hepatocytes kit, Stem Cell Technologies, Vancouver, BC, Canada). At the end of differentiation, cells were fixed with cold methanol (-20°C) and quickly washed with PBS. Membranes were permeabilized by Triton X100 (0.1%) in PBS-BSA (2%) for 10 minutes at room temperature and washed with PBS before adding blocking solution (PBS-BSA 2%) for 30 minutes at room temperature. The coverslips were incubated with primary antibodies (Supplementary Table S2) overnight at 4°C and the day after were washed with PBS. Secondary antibodies conjugated with Alexa-488 (Supplementary Table S3, Thermo Fisher Scientific) were incubated for 30 minutes at room temperature and then washed with PBS. Nuclei were counterstained with NucBlueTM Fixed Cell ReadyProbesTM Reagent (DAPI, Thermo Fisher Scientific). The coverslips were protected against fading with Vectashield® PLUS Antifade Mounting Medium (Vector Laboratories, H-1900) and images were acquired using confocal laser scanning microscopy Leica TCS SP5 II.

Supplementary Table 3: List of primary and secondary antibodies used for Western Blot (WB) and Immunofluorescence (IF) detection

**Protein Symbol**	**Species**	**Dilution and Company**
Calnexin (CANX)	Rabbit polyclonal	1:1000 WB, Enzo Life Science
Caspase 3	Rabbit polyclonal	1:1000 WB, Cell Signaling, clone D3R6Y)
Cleaved Caspase-3	Rabbit polyclonal	1:1000 WB, Cell Signaling, clone 5A1E
β-Actin	Mouse monoclonal	1:1000 Wb, Abcam, clone D6A8
GPR78	Rabbit polyclonal	1:1000 WB, Cell Signalling, clone C50B12
PARP	Rabbit polyclonal	1:1000 WB, Cell Signalling, clone 46D11
p16 INK4A	Rabbit polyclonal	1:1000 WB, Cell Signaling, clone D3W8G

p21 Waf1/Cip1	Rabbit monoclonal	1:1000 WB, Cell Signalling, clone 12D1
p53	Rabbit monoclonal	1:1000 WB, Cell Signalling, clone 7F5
HMGB1	Rabbit monoclonal	1:1000 WB, Cell Signalling, clone D3E5
iRhom2	Rat monoclonal	1:10 WB, Lichtenthaler lab [6]
Albumin	Mouse monoclonal	1:20 IF, R&D Systems
Cytokeratin-18	Rabbit monoclonal	1:50 IF, Abcam, clone EPR1626
α1-Antitrypsin	Rabbit monoclonal	1:100 IF, Abcam, clone EPR9090
IgG HRP conjugated	Anti-Rabbit	1:10000 WB, Promega
IgG HRP conjugated	Anti-Mouse	1:10000 WB, Promega
IgG (H+L) Highly Cross-Adsorbed, Alexa Fluor Plus 488	Goat anti-Rabbit	1:350 IF, Thermo Fischer Scientific
IgG (H+L) Highly Cross-Adsorbed, Alexa Fluor Plus 488	Goat anti-Mouse	1:350 IF, Thermo Fischer Scientific

References

1. Zito G, Miceli V, Carcione C, Busa R, Bulati M, Gallo A, Iannolo G, Pagano D, Conaldi PG: **Human Amnion-Derived Mesenchymal Stromal/Stem Cells Pre-Conditioning Inhibits Inflammation and Apoptosis of Immune and Parenchymal Cells in an In Vitro Model of Liver Ischemia/Reperfusion**. *Cells* 2022, **11**(4).
2. Calligaris M, Spano DP, Bonelli S, Muller SA, Carcione C, D'Apolito D, Amico G, Miele M, Di Bella M, Zito G *et al*: **iRhom2 regulates ectodomain shedding and surface expression of the major histocompatibility complex (MHC) class I**. *Cellular and molecular life sciences : CMLS* 2024, **81**(1):163.
3. Wisniewski JR, Zougman A, Nagaraj N, Mann M: **Universal sample preparation method for proteome analysis**. *Nature methods* 2009, **6**(5):359-362.
4. Rappsilber J, Ishihama Y, Mann M: **Stop and go extraction tips for matrix-assisted laser desorption/ionization, nanoelectrospray, and LC/MS sample pretreatment in proteomics**. *Analytical chemistry* 2003, **75**(3):663-670.
5. Tyanova S, Temu T, Sinitcyn P, Carlson A, Hein MY, Geiger T, Mann M, Cox J: **The Perseus computational platform for comprehensive analysis of (prote)omics data**. *Nature methods* 2016, **13**(9):731-740.
6. Weskamp G, Tushaus J, Li D, Feederle R, Maretzky T, Swendemann S, Falck-Pedersen E, McIlwain DR, Mak TW, Salmon JE *et al*: **ADAM17 stabilizes its interacting partner inactive Rhomboid 2 (iRhom2) but not inactive Rhomboid 1 (iRhom1)**. *The Journal of biological chemistry* 2020, **295**(13):4350-4358.
