# Supplementary figures and images for: iRhom2 regulates HMGB1 secretion to modulate inflammation and hepatocyte senescence in an in vitro model of ischemia-reperfusion injury
Source: Cell Death Dis. 2026 Jan 7;17(1):7. doi: 10.1038/s41419-025-08256-x (PMC12780076; doi:10.1038/s41419-025-08256-x)

**Figure 2**

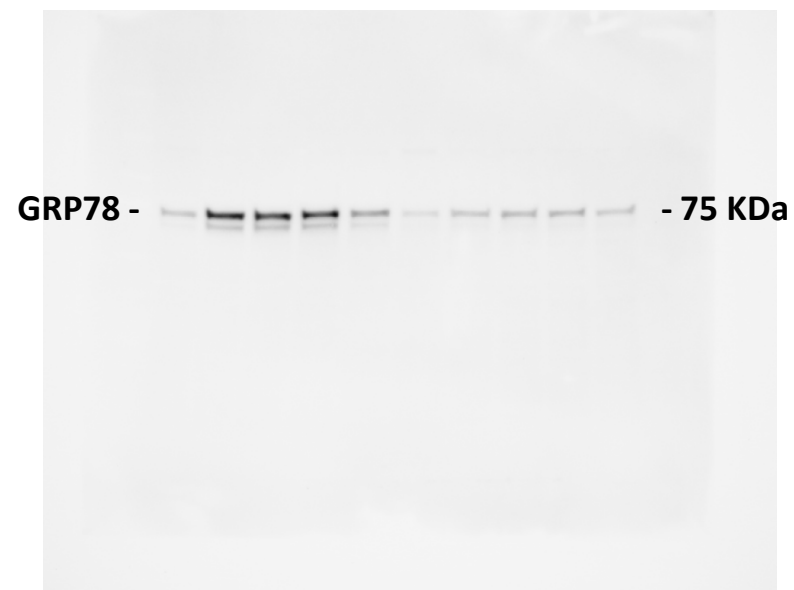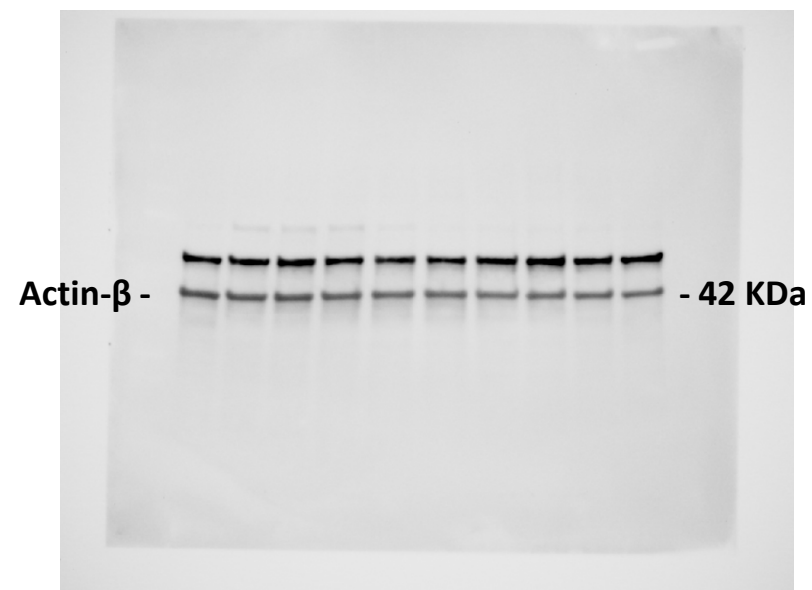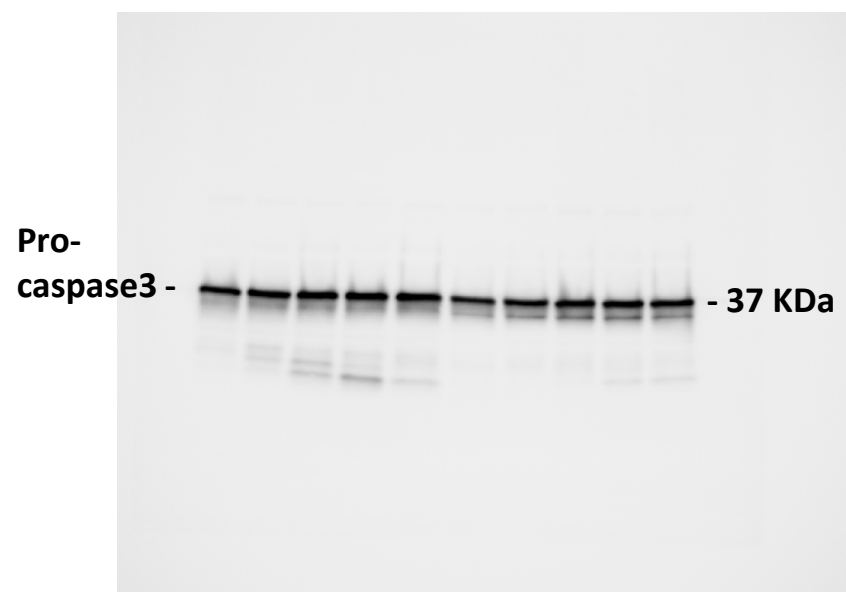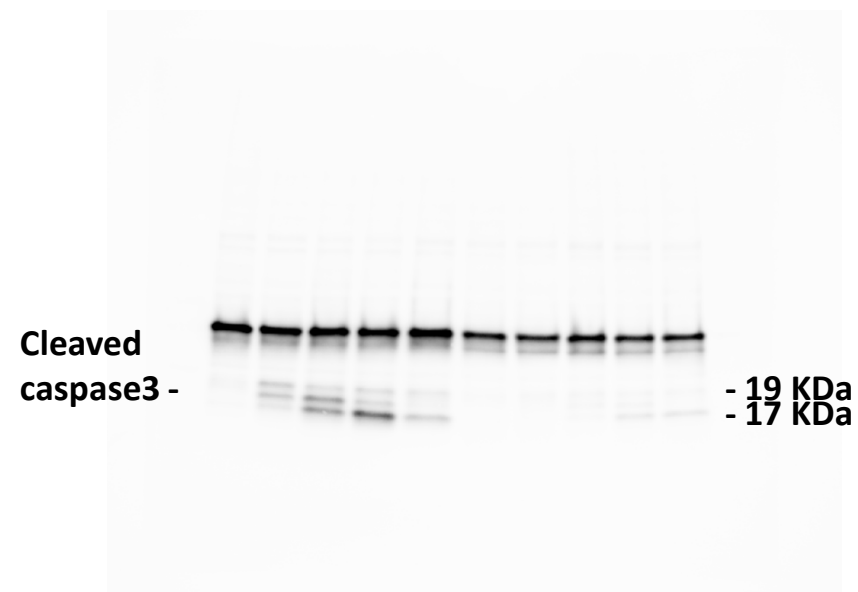

**Figure 2**

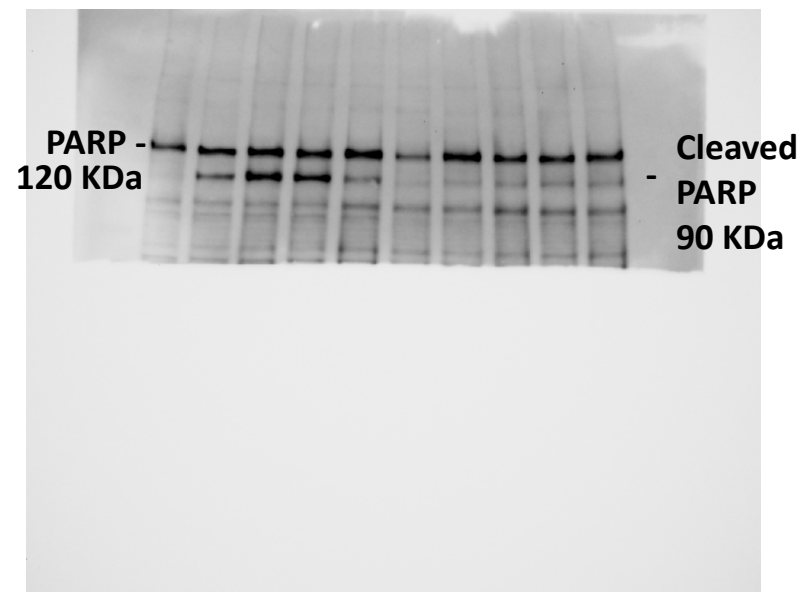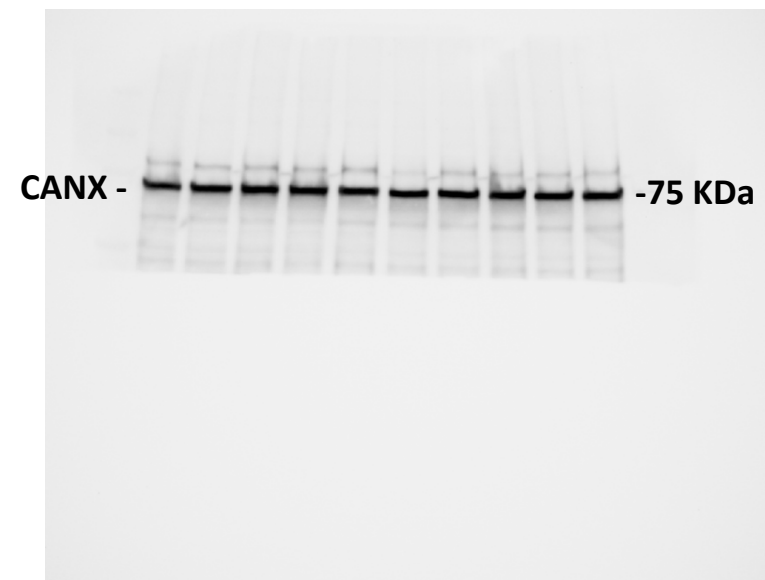

**Figure 5**

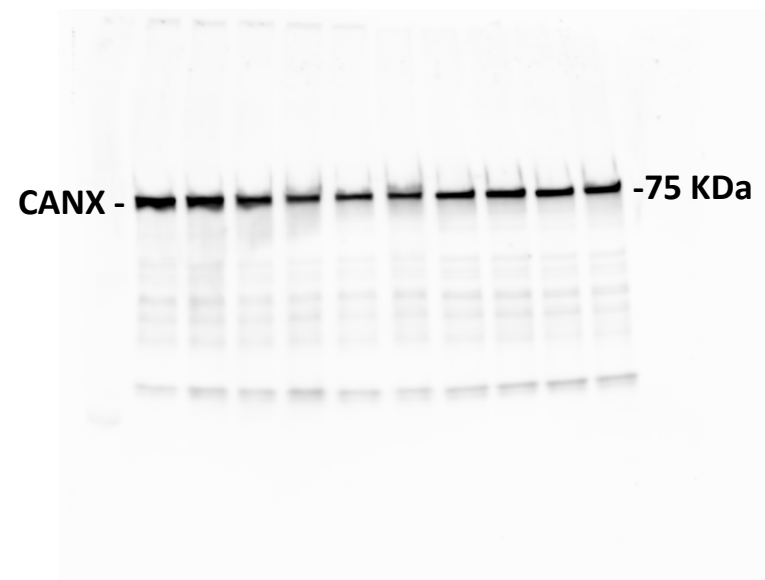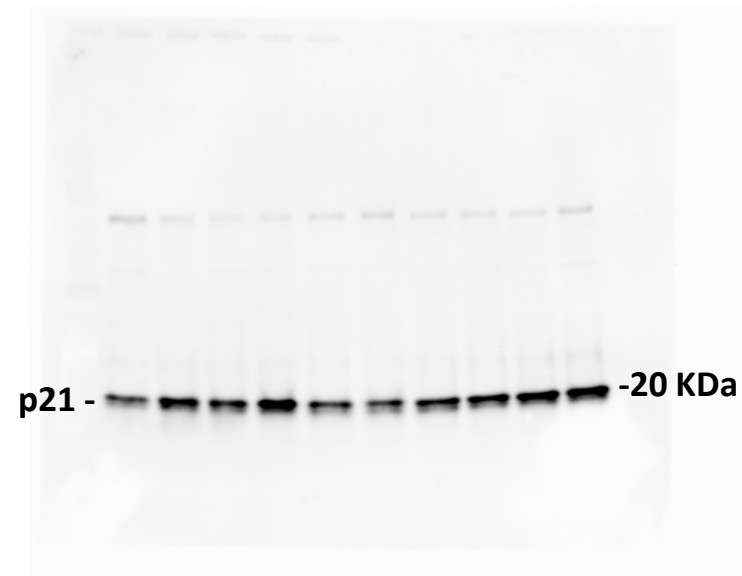

**Figure 6**

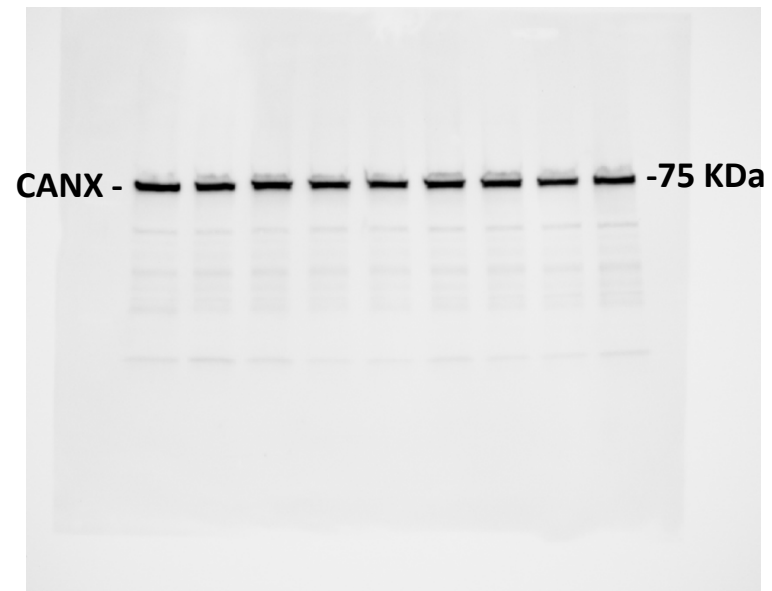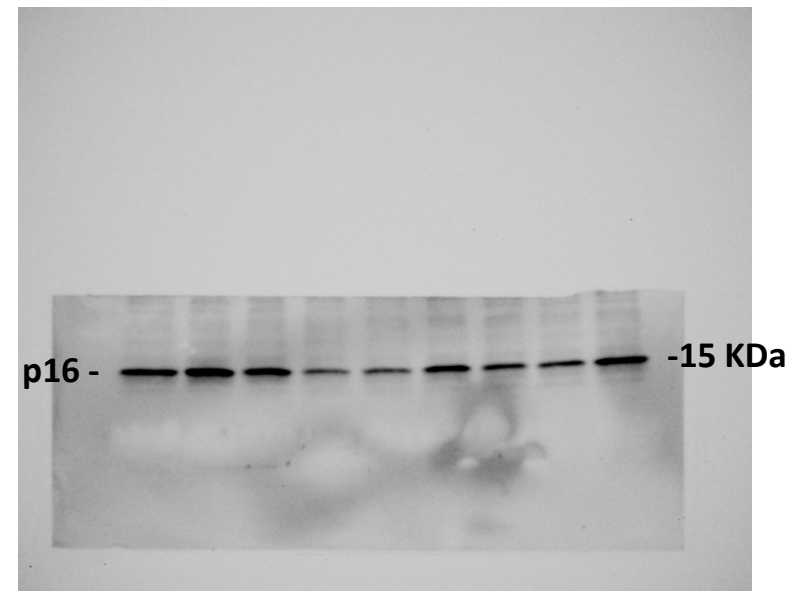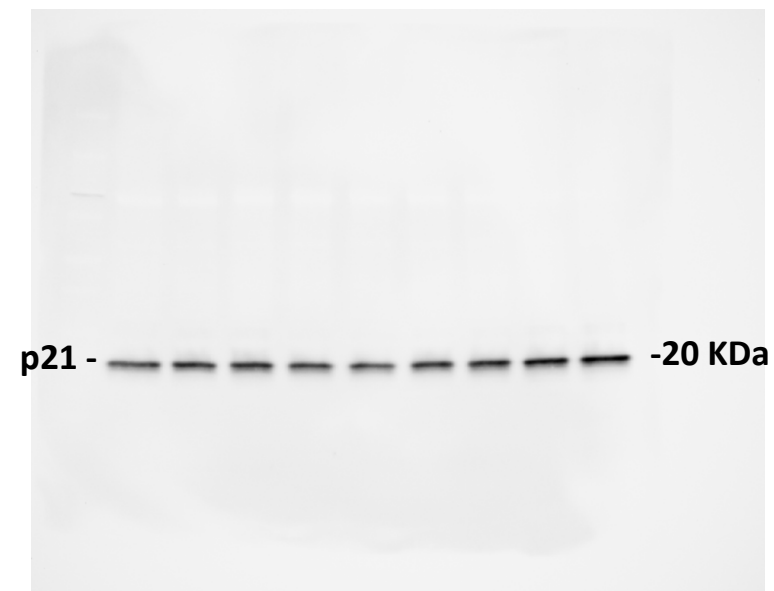

**Figure 6**

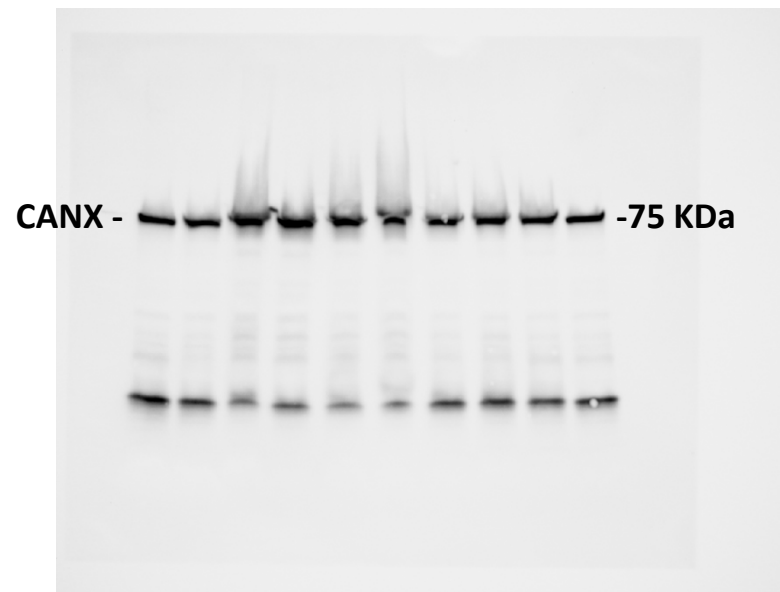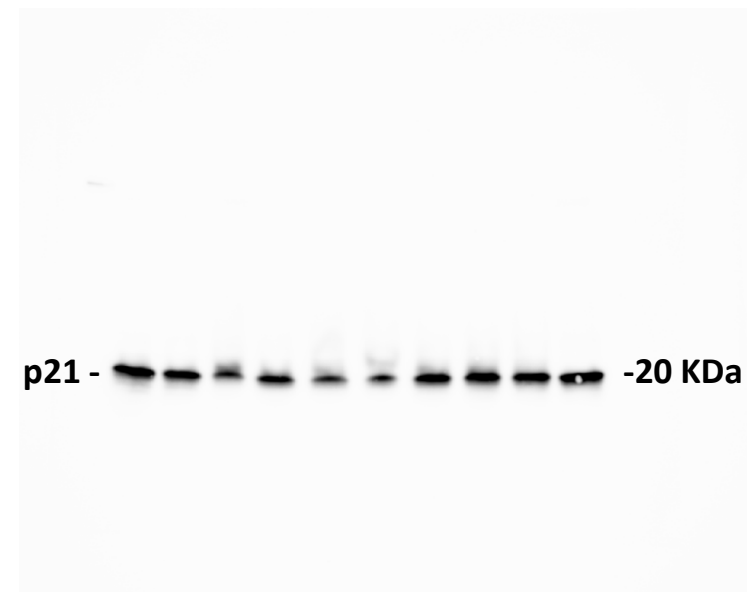

**Supplementary figure S4**

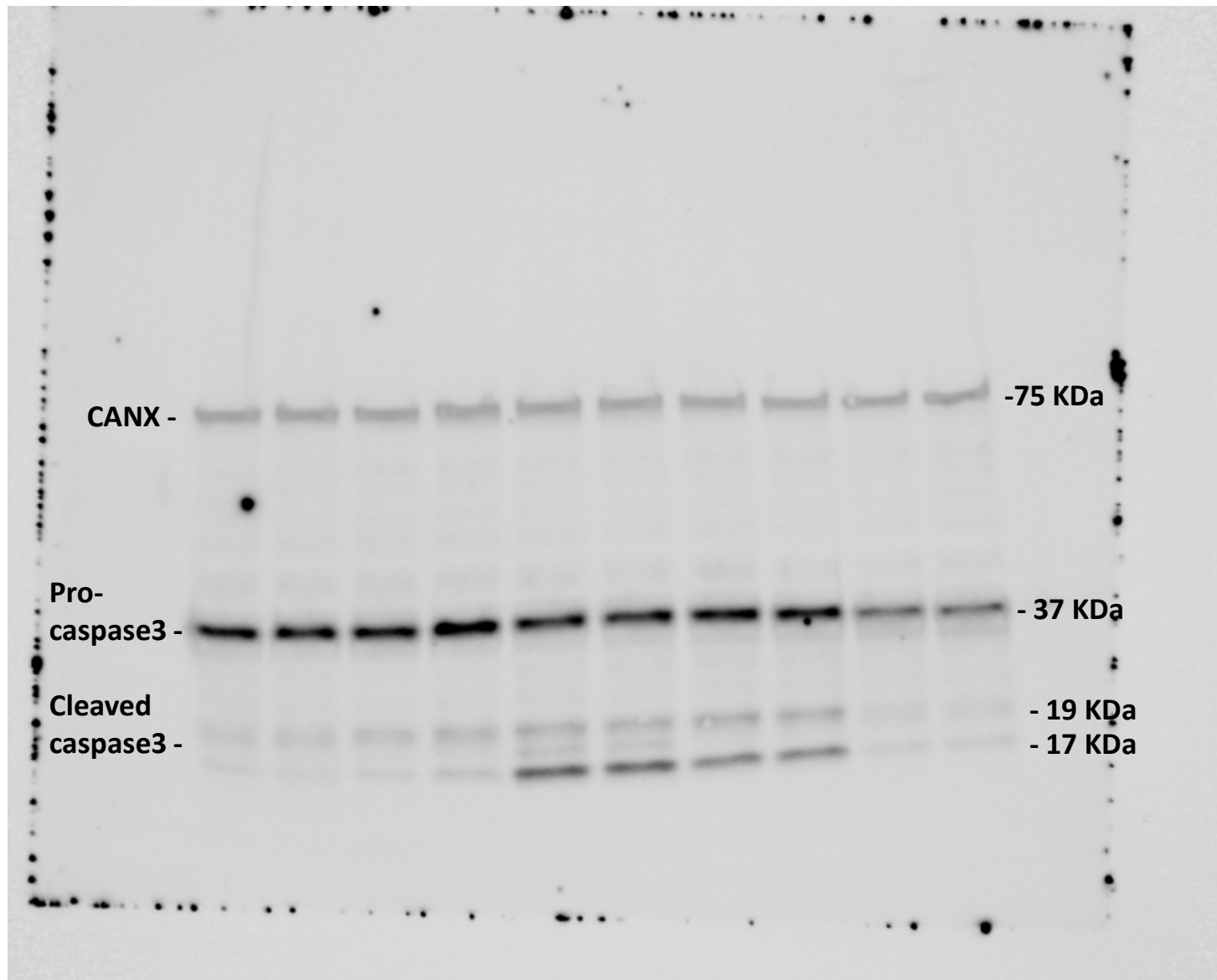

Supplement: Supplementary file 2 — Supplementary FIles_ Original WB [file 41419_2025_8256_MOESM2_ESM.pdf]
